# The Siderophore Ferricrocin Mediates Iron Acquisition in Aspergillus fumigatus

**DOI:** 10.1128/spectrum.00496-23

**Published:** 2023-05-18

**Authors:** Isidor Happacher, Mario Aguiar, Mostafa Alilou, Beate Abt, Tim J. H. Baltussen, Clemens Decristoforo, Willem J. G. Melchers, Hubertus Haas

**Affiliations:** a Institute of Molecular Biology, Biocenter, Medical University Innsbruck, Innsbruck, Austria; b Institute of Pharmacy, Unit of Pharmacognosy, Center for Molecular Biosciences Innsbruck, Austria; c Department of Medical Microbiology, Radboud University Medical Centre, Nijmegen, The Netherlands; d Centre of Expertise in Mycology, Radboudumc/CWZ, Nijmegen, The Netherlands; e Department of Nuclear Medicine, Medical University Innsbruck, Innsbruck, Austria; Septomics Research Center, Friedrich Schiller University and Leibniz Institute for Natural Product Research and Infection Biology-Hans Knöll Institute

**Keywords:** fungi, molds, *Aspergillus fumigatus*, iron, siderophore, ferricrocin, germination

## Abstract

The opportunistic fungal pathogen Aspergillus fumigatus utilizes two high-affinity iron uptake mechanisms, termed reductive iron assimilation (RIA) and siderophore-mediated iron acquisition (SIA). The latter has been shown to be crucial for virulence of this fungus and is a target for development of novel strategies for diagnosis and treatment of fungal infections. So far, research on SIA in this mold focused mainly on the hyphal stage, revealing the importance of extracellular fusarinine-type siderophores in iron acquisition as well as of the siderophore ferricrocin in intracellular iron handling. The current study aimed to characterize iron acquisition during germination. High expression of genes involved in biosynthesis and uptake of ferricrocin in conidia and during germination, independent of iron availability, suggested a role of ferricrocin in iron acquisition during germination. In agreement, (i) bioassays indicated secretion of ferricrocin during growth on solid media during both iron sufficiency and limitation, (ii) ferricrocin was identified in the supernatant of conidia germinating in liquid media during both iron sufficiency and limitation, (iii) in contrast to mutants lacking all siderophores, mutants synthesizing ferricrocin but lacking fusarinine-type siderophores were able to grow under iron limitation in the absence of RIA, and (iv) genetic inactivation of the ferricrocin transporter Sit1 decreased germination in the absence of RIA. Taken together, this study revealed that ferricrocin has not only an intracellular role but also functions as an extracellular siderophore to support iron acquisition. The iron availability-independent ferricrocin secretion and uptake during early germination indicate developmental, rather than iron regulation.

**IMPORTANCE** Aspergillus fumigatus is one of the most common airborne fungal pathogens for humans. Low-molecular-mass iron chelators, termed siderophores, have been shown to play a central role in iron homeostasis and, consequently, virulence of this mold. Previous studies demonstrated the crucial role of secreted fusarinine-type siderophores, such as triacetylfusarinine C, in iron acquisition, as well as of the ferrichrome-type siderophore ferricrocin in intracellular iron storage and transport. Here, we demonstrate that ferricrocin is also secreted to mediate iron acquisition during germination together with reductive iron assimilation. During early germination, ferricrocin secretion and uptake were not repressed by iron availability, indicating developmental regulation of this iron acquisition system in this growth phase.

## INTRODUCTION

Aspergillus fumigatus is a ubiquitously found fungal species living on dead organic material. Due to their small size, the airborne conidia produced by asexual sporulation are easily inhaled. Particularly in immunocompromised patients, this can potentially lead to invasive infections, termed invasive aspergillosis (1). This opportunistic fungal pathogen has developed mechanisms to cope with various stressors allowing human infection. These include strategies to acquire access to nutrients that are not readily available (1). The essential trace element iron plays an important role in the virulence of A. fumigatus, as adaptation to iron limitation is required during infection (2–5). Apart from being one of the most abundant metals in the Earth’s crust, iron has a very low bioavailability, as it easily reacts with oxygen, leading to hardly soluble hydroxides, which hinders iron acquisition also during saprobic growth (6).

**FIG 1:**
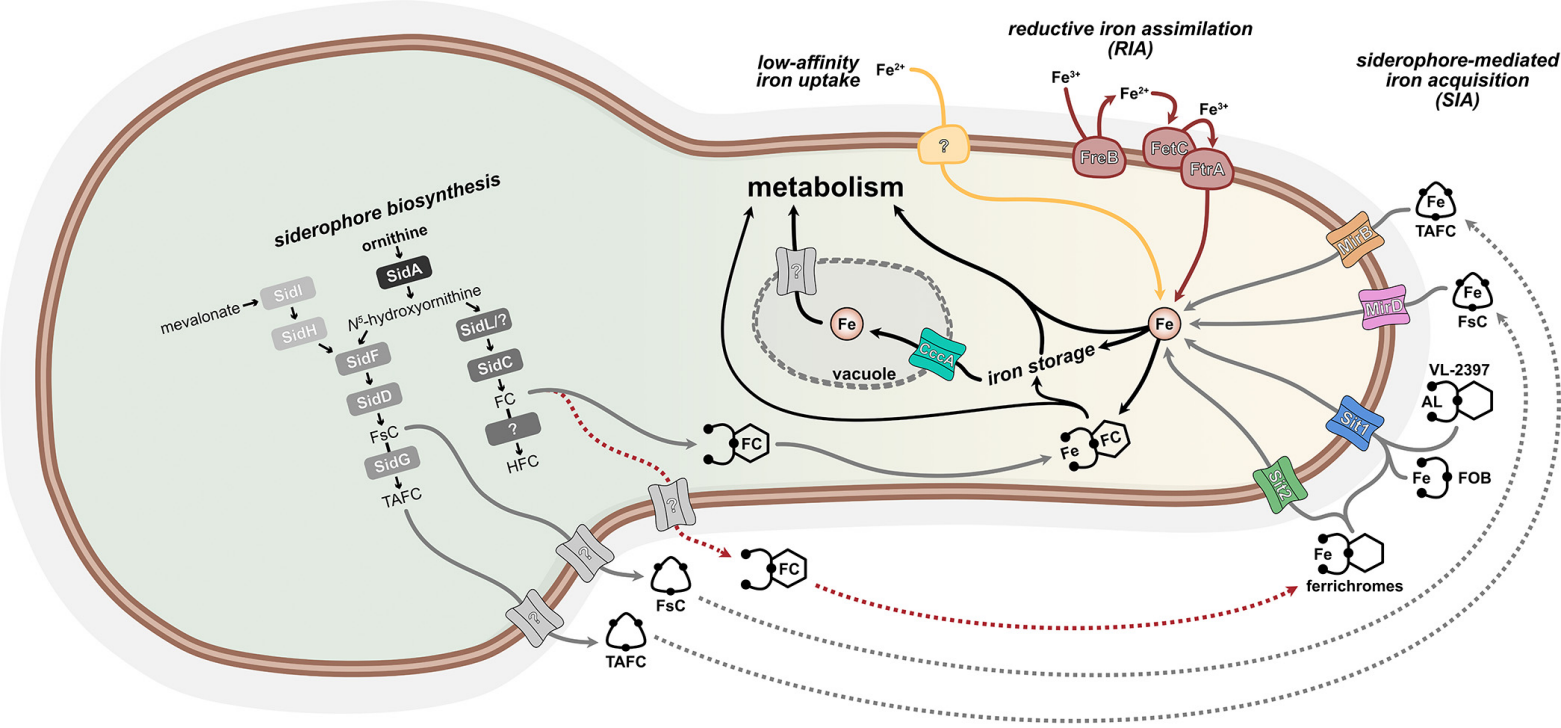
Schematic for siderophore biosynthesis and uptake in A. fumigatus. A detailed description is found in the text. The red dotted arrows illustrate the pathway identified in this study. FC, ferricrocin; FsC fusarinine C; HFC, hydroxyferricrocin; TAFC, triacetylfusarinine C.

A. fumigatus evolved three different iron uptake systems, low-affinity ferrous iron uptake and the two high-affinity iron uptake mechanisms, termed reductive iron assimilation (RIA) and siderophore-mediated iron acquisition (SIA) (6, 7). A schematic summarizing high-affinity iron acquisition by SIA and RIA is shown in Fig. 1. RIA proceeds in three main steps. In the first step, ferric iron is extracellularly reduced to ferrous iron by metalloreductases, such as FreB, that are localized in the plasma membrane (6, 8). Ferrous iron is then reoxidized to ferric iron by the multicopper oxidoreductase FetC, which is coupled with cellular uptake of iron(III) by the iron permease FtrA (6, 7). Siderophores are low-molecular-mass iron(III)-chelating molecules secreted by the fungus to bind iron for uptake by reabsorption of the siderophores-iron complex (6). A. fumigatus secretes two fusarinine-type siderophores, triacetylfusarinine C (TAFC) and fusarinine C, to capture environmental iron (6, 9, 10). Moreover, it employs two ferrichrome-type siderophores, ferricrocin (FC) and hydroxyferricrocin, for intracellular handling of iron (6, 9–11). The common initial step for biosynthesis of both fusarinine-type and ferrichrome-type siderophores, formation of *N*^5^-hydroxyornithine from ornithine, is catalyzed by the monooxygenase SidA (6, 7, 12). Thereafter, the pathways for biosynthesis of fusarinine-type and ferrichrome-type siderophores split. For synthesis of ferrichrome-type siderophores, *N*^5^-hydroxyornithine is acetylated to *N*^5^-acetyl-*N*^5^-hydroxyornithine by the transacetylase SidL and another yet-unknown enzyme (6, 13). In the following step, the nonribosomal peptide synthetase (NRPS) SidC assembles FC from three *N*^5^-acetyl-*N*^5^-hydroxyornithine, two glycine, and one serine residue (6). Hydroxyferricrocin is formed from FC by a single hydroxylation by an as-yet-unknown enzyme (6). For fusarinine-type siderophore production, the transacylase SidF transfers anhydromevalonyl-CoA to *N*^5^-hydroxyornithine (6, 9, 14). Anhydromevalonyl-CoA is derived from mevalonate by CoA-ligation and dehydration mediated by the mevalonyl-CoA ligase SidI and the mevalonyl-CoA hydratase SidH (9, 14, 15). The linkage of three *N*^5^-anhydromevalonyl-*N*^5^-hydroxyornithine by the NRPS SidD leads to fusarinine C and its TAFC triple *N*^2^-acetylation catalyzed by SidG results in TAFC (9, 15).

Biosynthesis of both fusarinine-type and ferrichrome-type siderophores has been shown to be crucial for virulence of A. fumigatus and other fungal species (6, 7, 9, 14). FC was found to mediate iron transport within hyphae, particularly to fuel conidiation, while hydroxyferricrocin is found exclusively in conidia in which hydroxyferricrocin-chelated iron constitutes the main conidial iron storage (6, 9, 11, 13, 16). Furthermore, the lack of intracellular siderophores has been shown to reduce resistance to oxidative stress in both hyphae and conidia (9, 11, 12, 17). These findings and the impact on fungal virulence highlight the role of intracellular iron handling by siderophores. However, hyphal iron detoxification was found to depend mainly on vacuolar iron deposition mediated by the transporter CccA, rather than FC-mediated iron chelation (18).

The task of extracellular siderophores is chelation of ambient iron for cellular uptake. Iron chelated by fusarinine C and TAFC is imported by the siderophore transporters MirD and MirB, respectively, and MirB has been shown to be crucial for virulence of A. fumigatus (19, 20). Notably, A. fumigatus employs two additional siderophore transporters, termed Sit1 and Sit2. Both accept ferrichrome-type siderophores, including FC as the substrate (10, 21). Sit1 additionally transports ferrioxamine-type siderophores (10, 21). Ferrioxamines are clearly xenosiderophores, as they are produced exclusively by bacterial species (10, 22, 23). If Sit1 and Sit2 are exclusively involved in uptake of ferrichrome-type siderophores secreted by other fungal species or if FC is secreted also by A. fumigatus is yet unresolved. Furthermore, Sit1 was shown to mediate uptake of the novel antifungal drug VL-2397 (formerly known as ASP2397), which has a ferrichrome-type structure (24). A fifth putative siderophore transporter, MirC, might play a function in FC biosynthesis (25).

In A. fumigatus, iron limitation causes transcriptional remodeling, including induction of high-affinity iron acquisition and downregulation of iron-consuming pathways (26, 27). The key regulators for maintaining iron homeostasis are the iron-sensing transcription factors SreA and HapX, which are linked by a negative transcriptional feedback loop (6, 26–28). SreA represses high-affinity iron uptake via both SIA and RIA during iron sufficiency. During iron limitation, HapX represses iron-consuming pathways, including vacuolar iron storage, and activates SIA and RIA. Under iron excess, however, HapX activates vacuolar iron deposition, which is important for iron detoxification (29).

Most previous studies on maintenance of iron homeostasis of A. fumigatus focused on the hyphal stage. The aim of the current study was the characterization of iron acquisition during conidial germination.

## RESULTS

### Genes involved in ferricrocin biosynthesis and uptake show high conidial transcript levels and high expression during germination.

**FIG 2:**
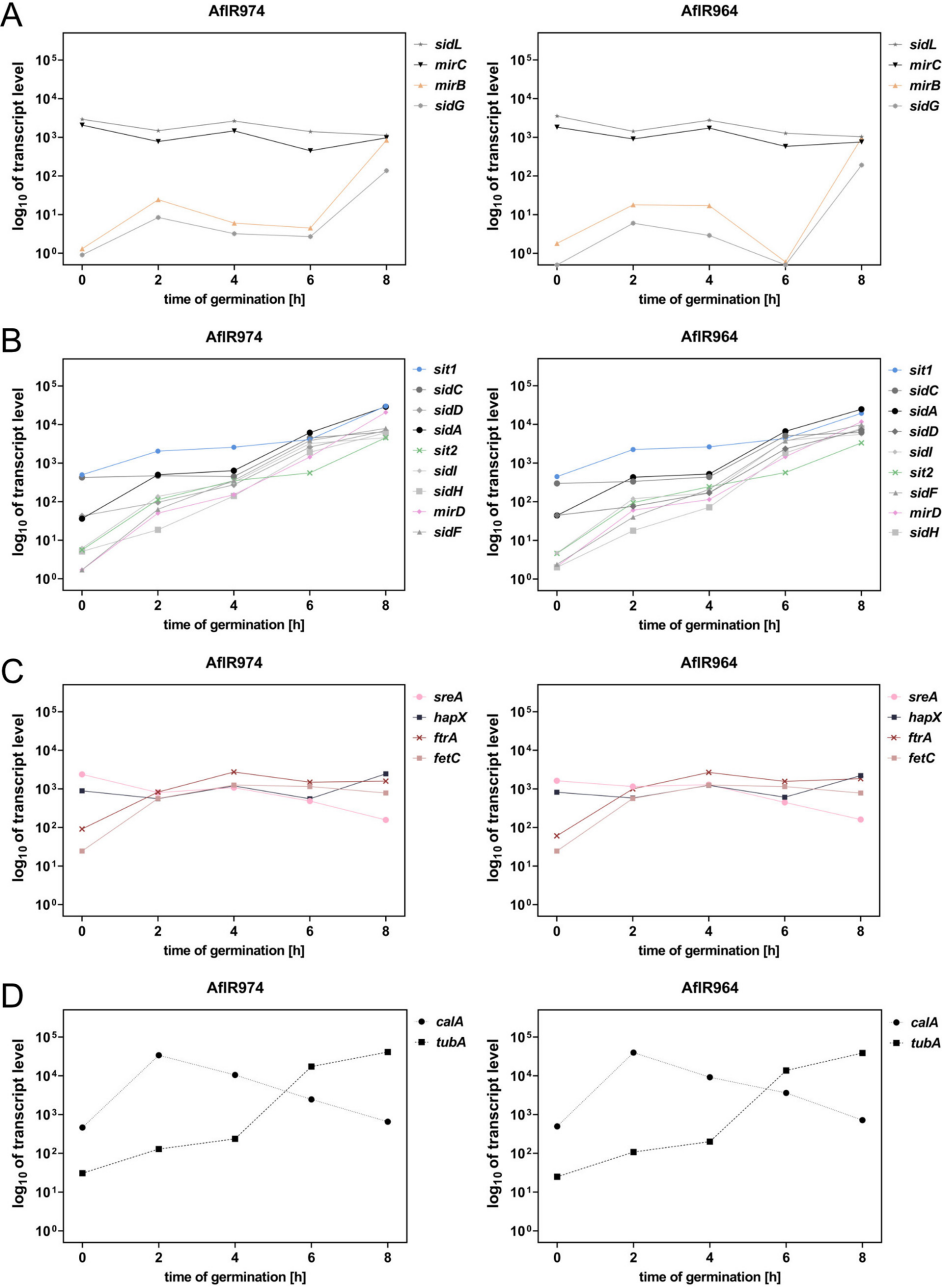
The transcript levels of most genes involved in SIA increase during the germination process. Shown are the mean values of two biological replicates of Aspergillus fumigatus strains AflR974 and AflR964, reanalyzed from Baltussen et al. (30). (A) *mirC*, *mirB*, *sidL*, and *sidG*. (B) *sit1*, *sit2*, *mirD*, *sidA*, *sidI*, *sidH*, *sidD*, *sidC*, and *sidF*. (C) *fetC*, *ftrA*, *hapX*, and *sreA*. (D) *calA* and *tubA*.

To investigate potential iron regulation at the transcriptional level during germination, the transcriptome data sets described by Baltussen et al. (30) were reanalyzed in this respect (Fig. 2). The transcript levels (TLs) of most characterized genes involved in SIA increased significantly during the 8-h germination process but showed different patterns, which were largely similar in both A. fumigatus strains analyzed, AfIR974 and AfIR964. Interestingly, the genes exclusively involved in TAFC metabolism (9, 20), TAFC transporter-encoding *mirB* (AFUA_3G03640) and the transacetylase-encoding *sidG* (AFUA_3G03650), showed very low TLs up to 6 h after initiation of germination before they significantly increased (Fig. 2A). *sidF* (AFUA_3G03400), *sidH* (AFUA_3G03410), *sidI* (AFUA_1G17190), and *mirD* (AFUA_3G03440), which are involved in biosynthesis and uptake of fusarinine-type siderophores (6, 9, 14, 20), had higher TLs in conidia (0-h time point), followed by a faster increase (Fig. 2B). Compared to these, *sidA* (AFUA_2G07680) and *sidD* (AFUA_3G03420) showed higher conidial TLs, while *sidC* (AFUA_1G17200) and *sit1* (AFUA_7G06060), which are involved in FC biosynthesis and uptake (6, 9, 10, 21, 24), displayed the highest conidial TLs (Fig. 2B).

In contrast, *mirC* (AFUA_2G05730), with unresolved function, and FC biosynthetic *sidL* (AFUA_1G04450) displayed high conidial TLs with minor fluctuations during germination (Fig. 2A) (6, 13, 25).

The iron transporter-encoding *ftrA* (AFUA_5G03800) and the multicopper oxidoreductase *fetC* (AFUA_5G03790), which are core components of RIA (6, 7), displayed a medium conidial TL and increased up to the 4 h of germination with minor fluctuation thereafter (Fig. 2C).

Iron regulatory transcription factor-encoding *sreA* (AFUA_5G11260) and *hapX* (AFUA_5G03920) (6, 26, 27) showed similar high conidial TLs with low fluctuation up to the 6-h time point (Fig. 2C). The 8-h time point, however, showed a decrease in *sreA* coupled with an increase of *hapX*. This inverse behavior might be explained by the described transcriptional feedback loop, indicating iron sensing at this time point (6, 26–28).

As controls, the expression of two further genes, *calA* (AFUA_3G09690) and *tubA* (AFUA_1G10910), was examined (Fig. 2D). Invasin-encoding *calA* (31) exhibited a high conidial TL with a sharp increase at the 2-h time point followed by a decline, which is in agreement with its previously reported germination-associated expression (32, 33). β-Tubulin-encoding *tubA* showed a medium conidial TL, which steadily increased up to the 8-h time point, similar to many siderophore metabolic genes (Fig. 2B). The increase in *tubA* expression during germination might indicate the increased demand for β-tubulin for cellular growth.

Taken together, these data indicate an increase of genes involved in SIA and RIA during the germination process, indicating an increased iron demand for growth. Interestingly, their increase within the first 6 h of germination was not paralleled by the TLs of iron regulator-encoding *sreA* and *hapX*. As the latter are known to be iron regulated at the TL level (6, 26, 27), the control of TLs of the RIA and SIA genes during germination appears to be mediated by developmental regulators rather than by the iron-sensing transcription factors. However, HapX was recently shown to be subject to posttranslational modification (34), and therefore, it cannot be excluded that SIA and RIA are influenced by posttranslational modification of HapX and/or SreA. Moreover, the significantly higher TLs of genes involved in biosynthesis and uptake of FC in conidia and early germination (*sit1*, *sidC*, *sidL*) than genes involved in biosynthesis and uptake of TAFC (*mirB*, *sidG*) indicated a role of biosynthesis and uptake of FC.

**FIG 3:**
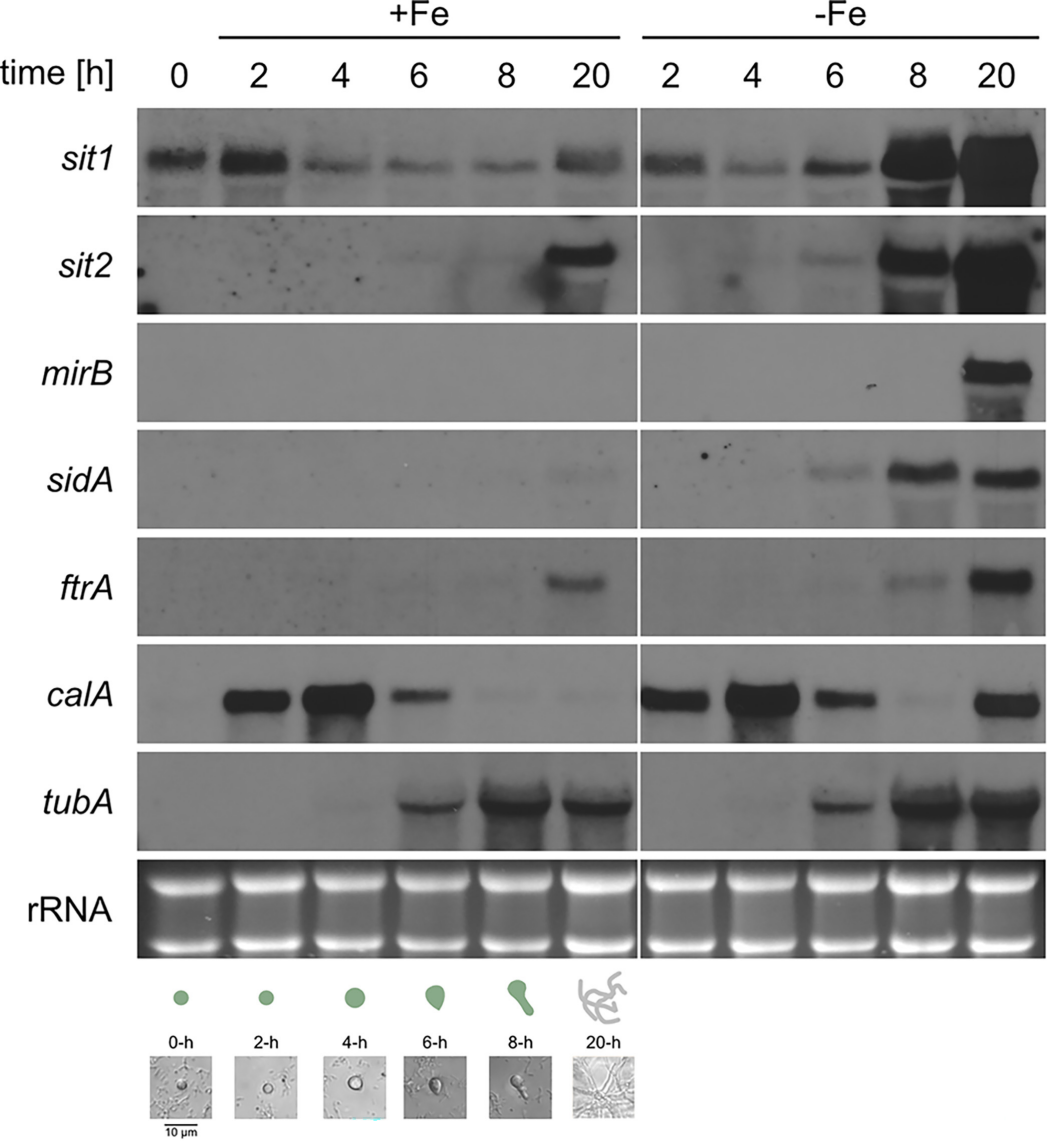
Compared to other investigated genes involved in SIA and RIA, *sit1* shows the highest transcript level in conidia and during gemination during both iron sufficiency and limitation. A. fumigatus WT was cultivated in Aspergillus minimal medium under iron limitation (−Fe) and iron sufficiency (+Fe; 0.03 mM FeSO_4_) for 0, 2, 4, 6, 8, and 20 h. The 50-mL liquid cultures inoculated with a total of 2.5 × 10^9^ spores were incubated at 37°C and shaken at 200 rpm. Northern blot analysis was performed with isolated total RNA. Ethidium bromide-stained rRNA serves as a control for loading and quality of the RNA. The pictures underneath the Northern blot analyses display the developmental stages, dormant conidia at 0 h; swelling, germ bud formation, and germling formation at about 4 h, 6 h, and 8 h, respectively; and the hyphal stage at 20 h after initiation of germination. Quantification of transcript levels is shown in Table S1 in the supplemental material.

The transcriptome studies by Baltussen et al. (30) were conducted in RPMI 1640 medium. To investigate the potential effect of environmental iron availability, the expression of selected genes during germination was analyzed by Northern blotting in minimal medium reflecting iron sufficiency (+Fe) and iron limitation (−Fe) using A. fumigatus strain AfS77, termed wild type (WT) (Fig. 3). Therefore, total RNA was isolated from dormant conidia (0 h) as well as 2 h, 4 h, 6 h, 8 h, and 20 h after initiation of germination. The 20-h time points served as control for the hyphal stage. In agreement with the transcriptome data, *sit1* showed a higher conidial transcript level than the other investigated genes (*sit2*, *mirB*, *sidA*, *ftrA*, *calA*, and *tubA*), as transcripts of these were not detected in this stage. During both iron sufficiency and limitation, *sit1* also displayed a high transcript level at the 2-h time point and decreased at the 4-h time point. During iron sufficiency, the 6-h and 8-h time points showed the same low transcript level as the 4-h time point, while a moderate increase was observed at the 20-h time point. In contrast, during iron limitation, the *sit1* transcript level increased slightly at the 6-h, massively at the 8-h, and even higher at the 20-h time point. In contrast to *sit1*, *sit2* transcript levels were detected during iron sufficiency only in the 20-h hyphal stage, most likely reflecting mild iron limitation, and during iron limitation starting weakly at the 6-h, followed by a massive increase at the 8-h and 20-h time points. Transcription of *mirB* was observed only at the 20-h hyphal stage during iron limitation. Transcription of *sidA* was detected during iron sufficiency at the 20-h time point and during iron limitation starting at the 6-h time point, followed by an increase at the 8-h and 20-h time points. Similarly, *ftrA* transcription was found during iron sufficiency at the 20-h time point and during iron limitation starting at the 8-h time point, followed by an increase at the 20-h time point. In agreement with previously reported expression, data, transcription of *calA* was not detected in conidia, but at the 2-h time point, followed by an increase at the 4-h time point and a decline at the 6-h time point, independent of iron availability. In line with this expression pattern, CalA was shown to be expressed on the surface of swollen conidia (31). Notably, in the 20-h hyphal stage, *calA* was found to be highly expressed during iron limitation but not sufficiency indicating a role of CalA in adaptation to iron limitation. In agreement with the transcriptome data, transcription of β-tubulin-encoding *tubA*, which is often used as a housekeeping control gene, was detected starting at the 6-h time point, followed by an increase at the 8-h time point, independent of iron availability.

Taken together, the Northern blot analysis largely confirmed the transcriptome data shown above. Of all genes involved in iron acquisition investigated in the Northern blot analyses, *sit1* has the highest transcript levels in conidia and during germination. The increased transcript levels of *sit1*, *sit2*, and *sidA* at the 6-h, and particularly the 8-h, time points during iron limitation compared to iron sufficiency indicate that sensing of environmental iron availability starts at this developmental stage. Consequently, the high transcript level of *sit1* in conidia and during germination might indicate iron availability-independent developmental regulation, as found for *calA* and *tubA*.

**FIG 4:**
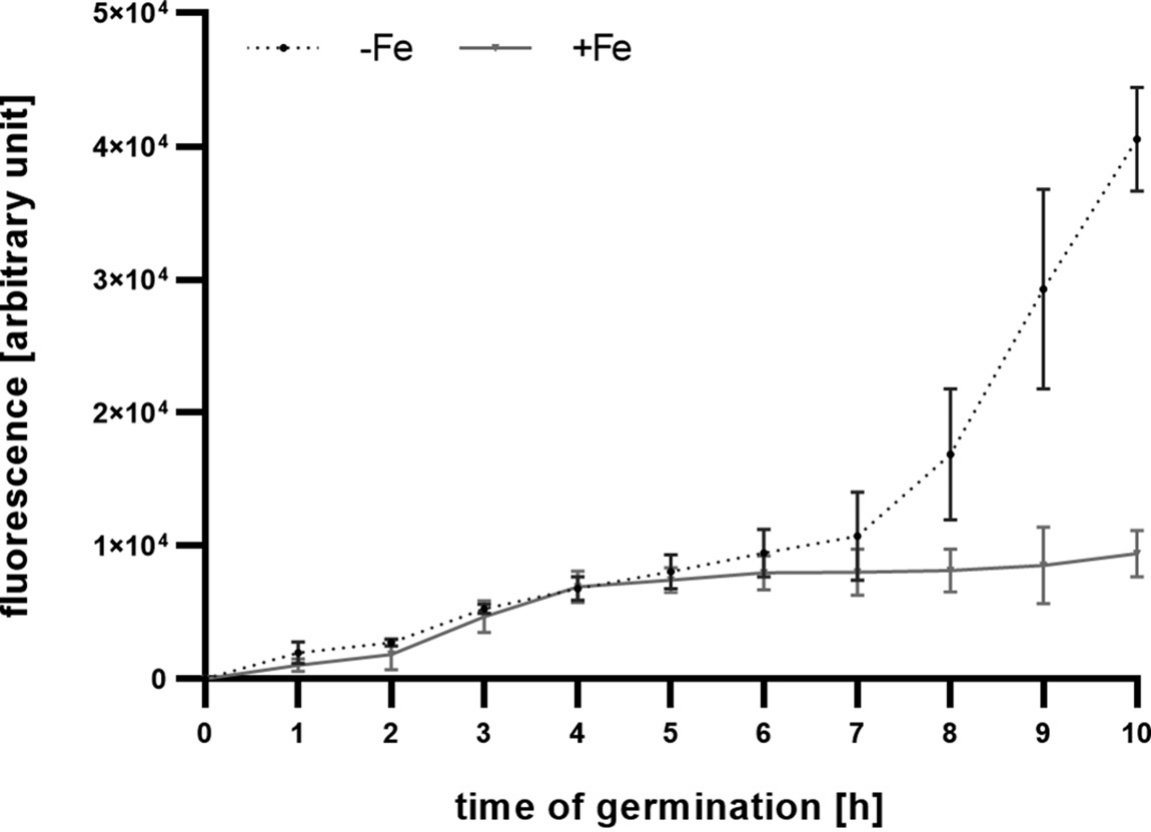
Fluorescence monitoring of a mutant strain producing Venus-tagged Sit1 demonstrates production of Sit1 protein during germination under both iron sufficiency and limitation. Aspergillus fumigatus WT and *sit1^C-Venus^* were grown in triplicates under iron limitation (−Fe) and iron sufficiency (+Fe; 0.03 mM FeSO_4_) conditions in Aspergillus minimal medium. Individual wells of a 96-well plate filled with 200 μL medium were inoculated with 5 × 10^6^ spores and then incubated to 37°C. Venus-mediated fluorescence was measured hourly over a period of 10 h using a fluorescence microplate reader. The graph shows the mean fluorescence with ± standard deviation (SD) of strain *sit1^C-Venus^* after subtraction of the background fluorescence of WT at the respective time point and of the 0-h time point.

Previous studies demonstrated that Sit1, C-terminally tagged with the yellow fluorescence protein derivative Venus and expressed under the control of the native promoter, localizes to the plasma membrane and functions WT-like with respect to transport of the ferrichrome-type compound VL-2397 (10, 24). Fluorescence monitoring of this strain demonstrated that production of the Sit1 protein increased similarly during iron limitation and sufficiency up to 6 h after initiation of germination (Fig. 4). Subsequently, production of Sit1 increased significantly more during iron limitation than sufficiency. Under iron sufficiency, the direct comparison of the *sit1* transcript level, which decreases after the 2-h time point (Fig. 3), and the fluorescence measurement-mediated Sit1 protein level, which continuously increases during germination (Fig. 4), shows a rather weak correlation. There are two not mutually exclusive explanations: (i) the transcript levels were normalized to rRNA and therefore roughly to the cell number, while the fluorescence measurements reflect a time course with increasing cell numbers during germination and hyphal elongation; and/or (ii) *sit1* transcript and Sit1 protein stability might significantly differ. Taken together, these data underline that Sit1 protein production increases during early germination, independent of environmental iron availability. The difference between iron sufficiency and limitation starting at about the 6-h time point matches the Northern blot results shown in Fig. 3.

### A. fumigatus secretes FC during growth in liquid and on solid media.

**FIG 5:**
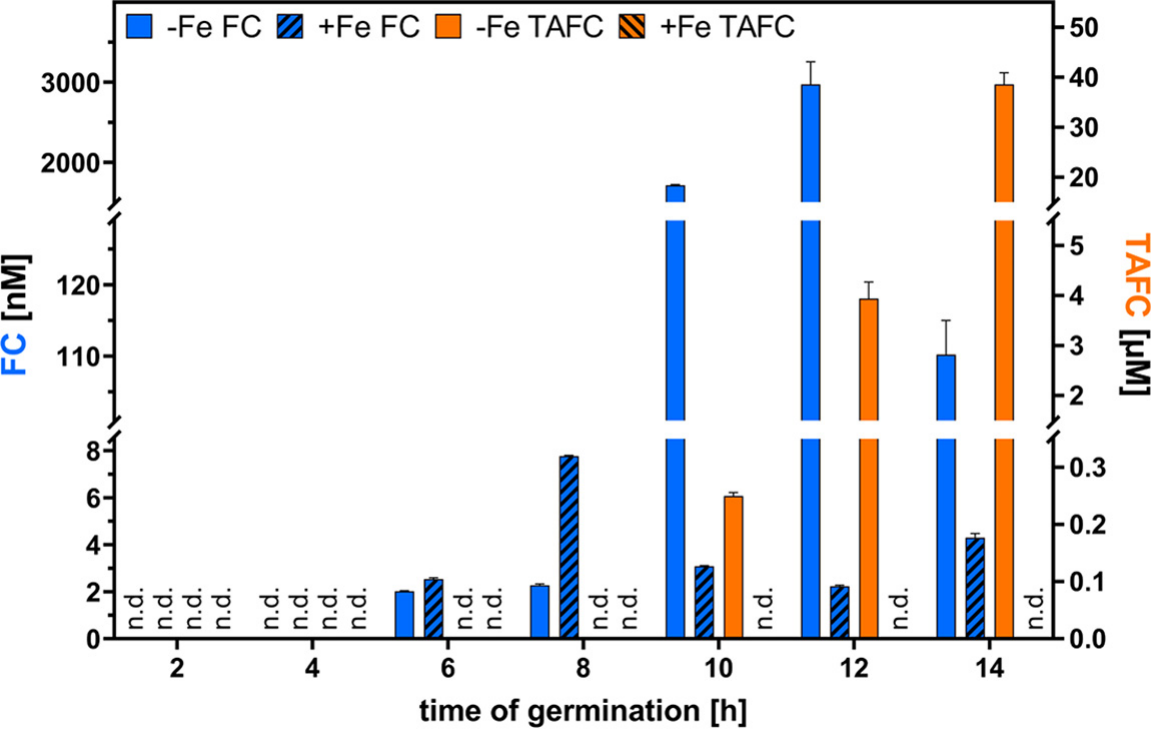
The supernatant of liquid-shake cultures of A. fumigatus contains FC during germination and hyphal growth under iron sufficiency and iron limitation, while TAFC is secreted exclusively during iron limitation starting in the hyphal stage. A. fumigatus WT was cultured as described in Fig. 3. After the respective time points, the supernatants were collected by filtration-mediated separation of the mycelia. Subsequently, siderophores were enriched by chromatography with Amberlite XAD-16N resin and elution with methanol followed by mass spectrometric analysis. The analysis shows the mean of three replicates ± SD. n.d., no siderophores detected.

The above-described observed high expression of *sit1* during germination indicated a role of FC uptake during germination, and thus, we analyzed if FC is secreted during germination. Indeed, FC quantification by mass spectrometric (MS) analyses identified FC in the supernatant of liquid-shake cultures starting 6 h after initiation of germination. The amount was similar during iron sufficiency and limitation at the 6-h time point and even 3.4 times higher at the 8-h time point during iron sufficiency than during iron limitation (Fig. 5), which indicates that FC secretion is not subject to iron repression at these time points. At later time points, extracellular FC displayed 25- to 1,330-fold-larger amounts during iron limitation than during sufficiency, suggesting that FC secretion is repressed by iron in the hyphal stage. The maximum extracellular FC concentration found was about 3 μM during iron limitation 12 h after initiation of germination, with a subsequent sharp decrease to 0.110 μM at the 14-h time point. Remarkably, TAFC was detected exclusively during iron limitation starting at the 10-h time point (Fig. 5), i.e., 4 h after FC secretion. In agreement with the transcriptome data and Northern blot analysis, these data indicate a role of FC, rather than TAFC, in germination.

**FIG 6:**
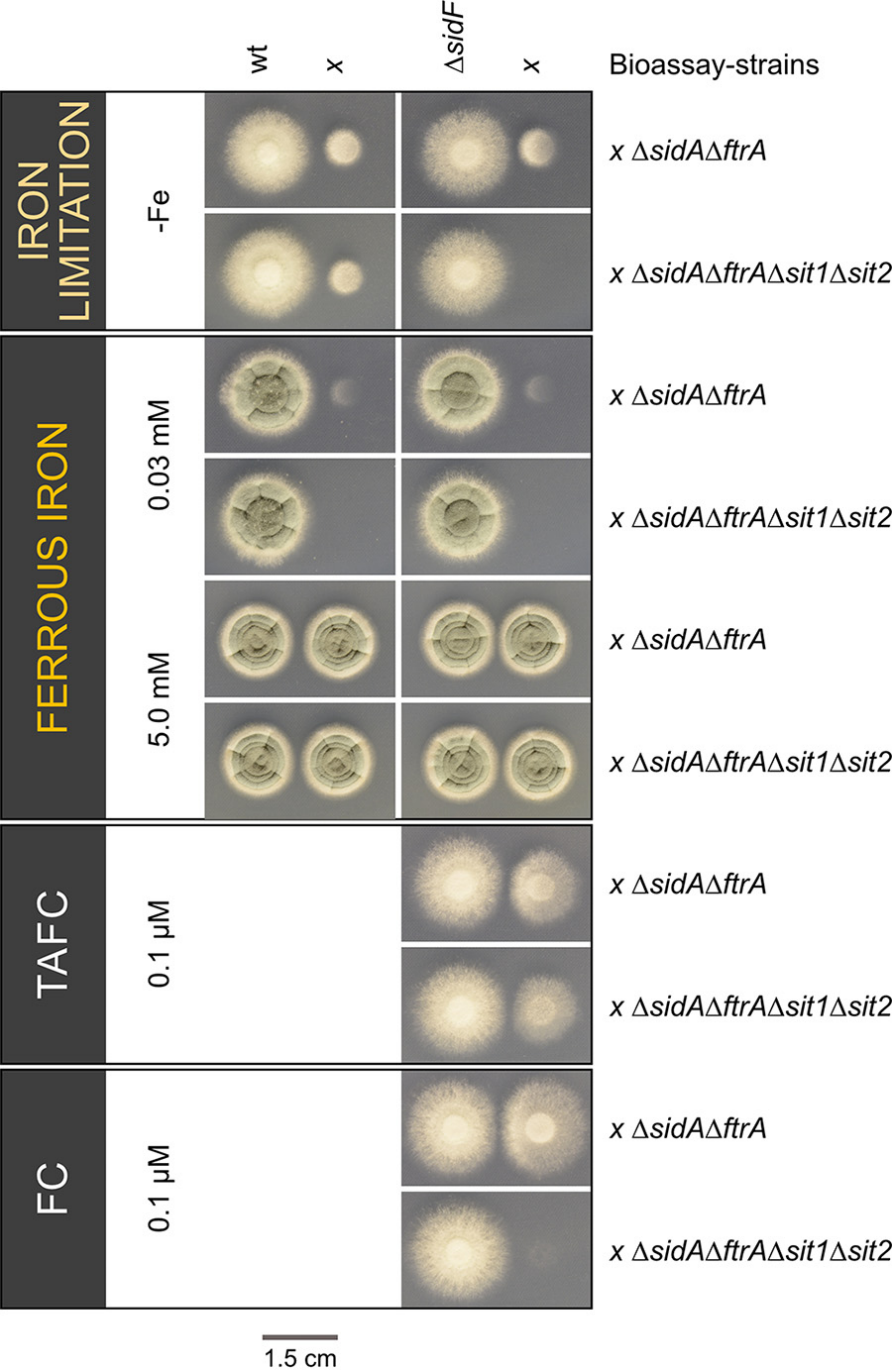
A. fumigatus secretes FC during growth on solid medium during both iron sufficiency and iron limitation. The Aspergillus fumigatus WT and Δ*sidF* mutant strains were point inoculated at a distance of 1.5 cm next to the bioassay strains Δ*sidAΔftrA* and Δ*sidAΔftrAΔsit1Δsit2*, using 10^4^ spores for all fungal strains. The growth conditions included iron limitation (−Fe), iron sufficiency (0.03 mM FeSO_4_), and iron excess with a concentration of 5.0 mM FeSO_4_. As a growth control, the bioassay strains were spotted next to Δ*sidF* on plates containing 0.1 μM TAFC or 0.1 μM FC. Plates were incubated at 37°C for 48 h.

Liquid-shake cultures have the advantage of easy separation of hyphae and culture supernatant, allowing state-of-the-art biochemical analyses. However, liquid-shake cultures might result in slight mechanical shearing, leading to cell lysis and subsequent release of intracellular metabolites such as FC. To avoid this potential problem, we analyzed, in the next step, FC secretion by fungal colonies grown on solid minimal medium using a bioassay employing the previously described A. fumigatus mutant strains Δ*sidAΔftrA* and Δ*sidAΔftrAΔsit1AΔsit2* (10). The Δ*sidAΔftrA* mutant lacks RIA (Δ*ftrA*) and siderophore biosynthesis (Δ*sidA*) and can consequently grow only in the presence of ferrous iron in concentrations >3 mM or siderophores such as FC and TAFC (7, 10, 35). The Δ*sidAΔftrAΔsit1AΔsit2* mutant shows a similar growth behavior but cannot utilize FC due to the additional absence of the two siderophore transporters Sit1 and Sit2 (10). Consequently, the growth of Δ*sidAΔftrA* indicates the presence of siderophores that are utilized by A. fumigatus, and the concomitant absence of growth of Δ*sidAΔftrAΔsit1AΔsit2* indicates that this siderophore is FC. The two bioassay strains were point inoculated at a distance of 1.5 cm of the A. fumigatus wild-type and the Δ*sidF* mutant strains on plates reflecting iron limitation (−Fe), iron sufficiency (0.03 mM FeSO_4_), and iron excess (5.0 mM FeSO_4_) (Fig. 6). The Δ*sidF* mutant strain was included, as it lacks production of fusarinine-type siderophores, which constitute the main extracellular siderophores in the hyphal stage (9).

Under iron limitation, the WT promoted growth of both bioassay strains in its vicinity, proving secretion of siderophores that consist not exclusively of FC. In agreement, the WT was shown to secrete the fusarinine-type siderophores fusarinine C and TAFC (9). Under iron sufficiency, however, the WT promoted growth of Δ*sidAΔftrA* but not of Δ*sidAΔftrAΔsit1AΔsit2*, indicating secretion of FC but not fusarinine-type siderophores. The sickle-shaped growth phenotype of Δ*sidAΔftrA* underlined the cross-feeding by siderophore diffusion from the WT side. Compared to iron-limiting conditions, the growth promotion was lower under iron sufficiency, which is in agreement with repression of production of fusarinine-type siderophores by iron. Nevertheless, these results indicated exclusive FC secretion by WT during iron sufficiency, while no conclusion regarding FC secretion during iron limitation can be drawn from this assay due to the secretion of fusarinine-type siderophores under this condition. Therefore, we analyzed siderophore secretion by the A. fumigatus Δ*sidF* mutant strain, which lacks biosynthesis of fusarinine-type siderophores and consequently produces exclusively ferrichrome-type siderophores (9). Under both iron limitation and sufficiency, the Δ*sidF* mutant promoted growth of Δ*sidAΔftrA* but not of Δ*sidAΔftrAΔsit1AΔsit2*, indicating secretion of FC. Remarkably, the growth promotion was higher during iron limitation, indicating that iron limitation increases FC secretion, which is in line with the recorded FC content of liquid culture supernatants starting at the 10-h time point (Fig. 5). A control experiment confirmed that both Δ*sidAΔftrA* and Δ*sidAΔftrAΔsit1AΔsit2* strains, spotted next to Δ*sidF*, grow on plates containing 5 mM FeSO_4_ or 0.1 μM TAFC, while only Δ*sidAΔftrA*, but not Δ*sidAΔftrAΔsit1AΔsit2*, was able to grow on plates containing 0.1 μM FC.

### Ferricrocin mediates high-affinity iron acquisition.

**FIG 7:**
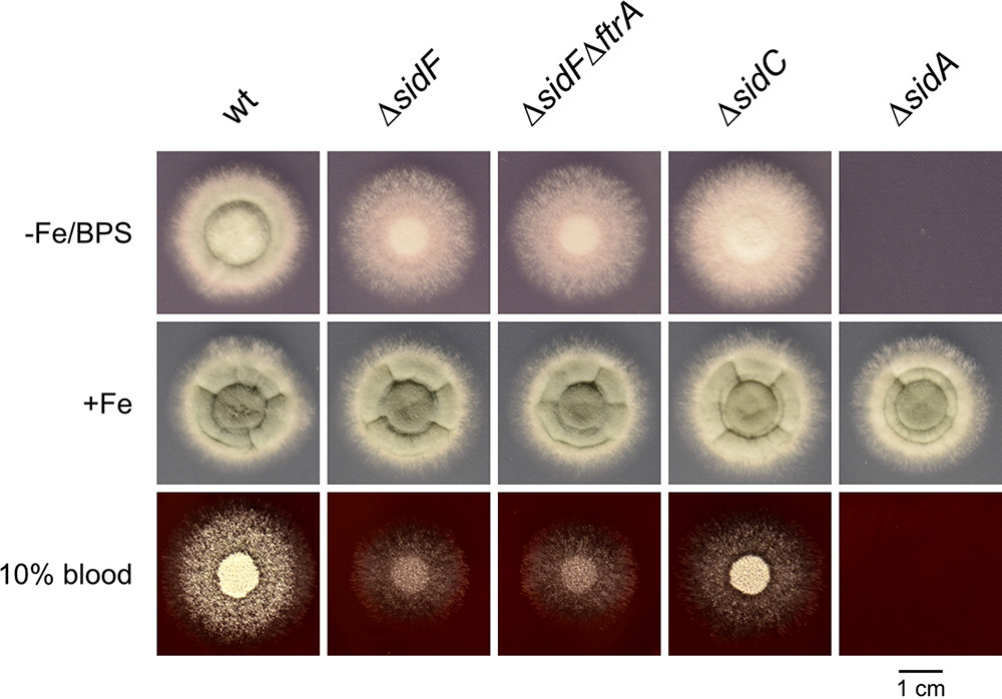
Lack of fusarinine-type siderophores (Δ*sidF* mutant) does not block growth of A. fumigatus under iron limitation and BPS-mediated inhibition of RIA and blood agar. We point inoculated 10^4^ conidia of Aspergillus fumigatus WT and Δ*sidF*, Δ*sidFΔftrA*, Δ*sidC*, and Δ*sidA* mutant strains on iron-depleted Aspergillus minimal medium (AMM) containing 150 μM BPS (−Fe/BPS), AMM containing 0.03 mM FeSO_4_ (+Fe), and 10% blood agar plates. Plates were incubated at 37°C for 48 h.

To analyze the potential impact of FC in iron acquisition, the growth of A. fumigatus strains with different defects in siderophore metabolism was compared to WT on solid medium reflecting iron limitation and containing the ferrous iron-specific chelator bathophenanthrolinedisulfonic acid (BPS), which inhibits siderophore-independent high-affinity iron acquisition mediated by RIA (7, 9). Consequently, only strains employing siderophore-mediated iron acquisition are able to grow under this condition. In agreement, the WT, but not the siderophore-lacking Δ*sidA* mutant strain, was able to grow under this condition (Fig. 7). Also, the Δ*sidC* mutant strain, which lacks ferrichrome-type siderophores but produces the major extracellular siderophores fusarinine C and TAFC (7, 9), was able to grow under this condition. Remarkably, the Δ*sidF* and Δ*sidFΔftrA* mutant strains were also able to grow in the presence of BPS (Fig. 7). The Δ*sidF* mutant strain produces exclusively ferrichrome-type siderophores such as FC, as it lacks biosynthesis of fusarinine-type siderophores, i.e., the two major extracellular siderophores fusarinine C and TAFC (9). These results indicate that ferricrocin mediates high-affinity iron uptake, which is in line with its secretion and upregulation of Sit1 expression during germination (Fig. 2 to 5). The similar growth patterns of Δ*sidF* and Δ*sidFΔftrA* underline that this growth condition inhibits RIA, which is defective in Δ*sidFΔftrA*, but not Δ*sidF*. Under iron sufficiency without BPS, all strains displayed similar radial growth, which demonstrates that the observed growth defects of certain strains are dependent on iron availability. Of note, all the strains analyzed exhibited a growth pattern on blood agar plates that largely resembled that on BPS plates; i.e., in contrast to Δ*sidA*, Δ*sidF,* and Δ*sidFΔftrA* mutant strains were able to grow, but not as well as Δ*sidC* and particularly WT. These data indicate that FC secretion might also impact pathogenicity of A. fumigatus.

Notably, Δ*ftrA*, Δ*sit1Δsit2*, and Δ*sit1Δsit2ΔftrA* mutants display WT-like radial growth on solid media (see Fig. S1A in the supplemental material) and biomass formation in liquid media (Fig. S1B), indicating that the combined lack of Sit1, Sit2, and RIA is compensated by iron uptake mediated by fusarinine C and/or TAFC. The phenotype of Δ*sidC* is therefore caused by the lack of intracellular functions of FC and/or hydroxyferricrocin (9, 11, 12, 17).

**FIG 8:**
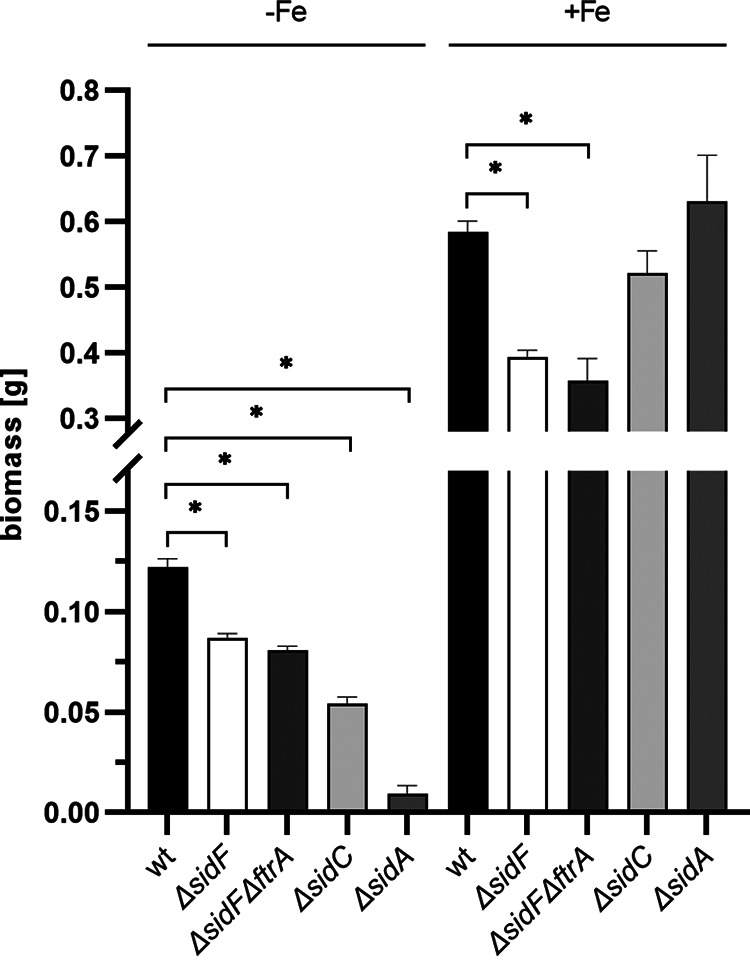
Lack of fusarinine-type siderophores (Δ*sidF* mutant) does not block growth of A. fumigatus under iron limitation in liquid cultures. A total of 10^8^ conidia of Aspergillus fumigatus WT and Δ*sidF*, Δ*sidFΔftrA*, Δ*sidC*, and Δ*sidA* mutant strains were cultivated in 100 mL AMM both under iron limitation (−Fe) and iron sufficiency (+Fe; 0.03 mM FeSO_4_) at 37°C for 20 h and shaken at 200 rpm. Shown are the mean values ± SD of biological triplicates; statistically significant differences by one-way analysis of variance (ANOVA) followed by Dunnett's multiple-comparison test are indicated by an asterisk (*P* ≤ 0.001).

To further analyze the impact of FC secretion on iron acquisition, growth of WT, Δ*sidF*, Δ*sidFΔftrA*, Δ*sidC*, and Δ*sidA* strains was compared during iron limitation and iron sufficiency in liquid-shake cultures (Fig. 8). During iron limitation, WT showed the highest and Δ*sidA* the lowest biomass formation, which largely matches growth on solid media with BPS or blood as a nutrient source. The Δ*sidF* and Δ*sidFΔftrA* showed similar growth, significantly lower than WT but significantly higher than Δ*sidA*, which confirms the role of FC in iron acquisition. Interestingly, Δ*sidC* showed lower growth than Δ*sidF* and Δ*sidFΔftrA*, which contrasts the growth phenotype of these mutants on solid media with BPS or blood (Fig. 7). Under iron sufficiency, biomass formation of all strains was significantly higher under iron limitation. WT, Δ*sidC*, and Δ*sidA* showed similar biomass formation, while Δ*sidF* and Δ*sidFΔftrA* displayed decreased biomass formation, which indicates that production of fusarinine-type siderophores is required for full biomass production under iron sufficiency.

### Sit1 plays a crucial role in germination.

**FIG 9:**
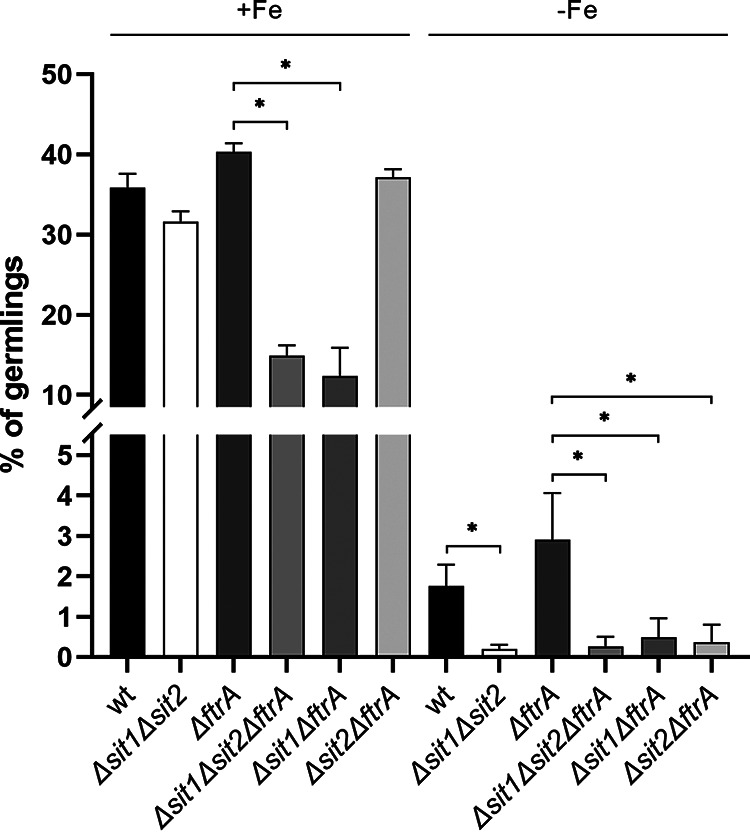
Sit1 plays a role in germination efficiency of Aspergillus fumigatus in the absence of RIA. We inoculated 1 × 10^5^ conidia of A. fumigatus WT and mutant strains in 0.5 mL minimal medium reflecting iron sufficiency (+Fe; 0.03 mM FeSO_4_) in a 24-well plate and incubated them at 37°C for 8 h. Subsequently, five random pictures of each well were taken, and all conidia and germlings were counted. Shown are the mean values ± SDs of biological triplicates. Statistically significant differences by one-way ANOVA followed by Dunnett's multiple-comparison test are indicated by an asterisk (*P* ≤ 0.001).

To investigate the role of FC uptake during germination, the germination rate of A. fumigatus WT and mutant conidia lacking Sit1 (Δ*sit1*), Sit2 (Δ*sit2*), or both (Δ*sit1Δsit2*) 8 h after inoculation of conidia into liquid medium was scored in the presence or absence (Δ*ftrA*) of RIA to decrease redundancy in high-affinity iron acquisition (Fig. 9). Under iron sufficiency, simultaneous loss of both Sit1 and Sit2 (Δ*sit1Δsit2*) resulted in a slightly decreased germination rate 8 h after initiation of germination compared to WT, although this was not statistically significant. In the absence of RIA, however, loss of Sit1 (Δ*sit1ΔftrA*) or simultaneous loss of both Sit1 and Sit2 (Δ*sit1Δsit2ΔftrA*), but not loss of Sit2 (Δ*sit2ΔftrA*) alone, resulted in a significantly reduced germination rate. Taken together, these data indicate that germination is fueled by both RIA and FC-mediated iron uptake via Sit1. Iron limitation reduced the germination rate compared to iron sufficiency. Under this condition, simultaneous loss of both Sit1 and Sit2 (Δ*sit1Δsit2*) reduced the germination rate compared to WT. In the absence (Δ*ftrA*) of RIA, loss of either Sit1 (Δ*sit1ΔftrA*) or Sit2 (Δ*sit2ΔftrA*) resulted in a significantly reduced germination rate. These data indicate that RIA has a lower impact on germination efficiency and that Sit2 becomes more important under iron limitation.

Of note, Δ*sit1Δsit2*, Δ*sit1Δsit2ΔftrA*, Δ*ftrA*, and WT display similar radial growth on solid media (Fig. S1A) and biomass formation in liquid media (Fig. S1B), indicating that the mutants recover despite the initial germination delay.

### FC promotes colony formation by A. fumigatus Δ*sidAΔftrA* earlier than TAFC.

**FIG 10:**
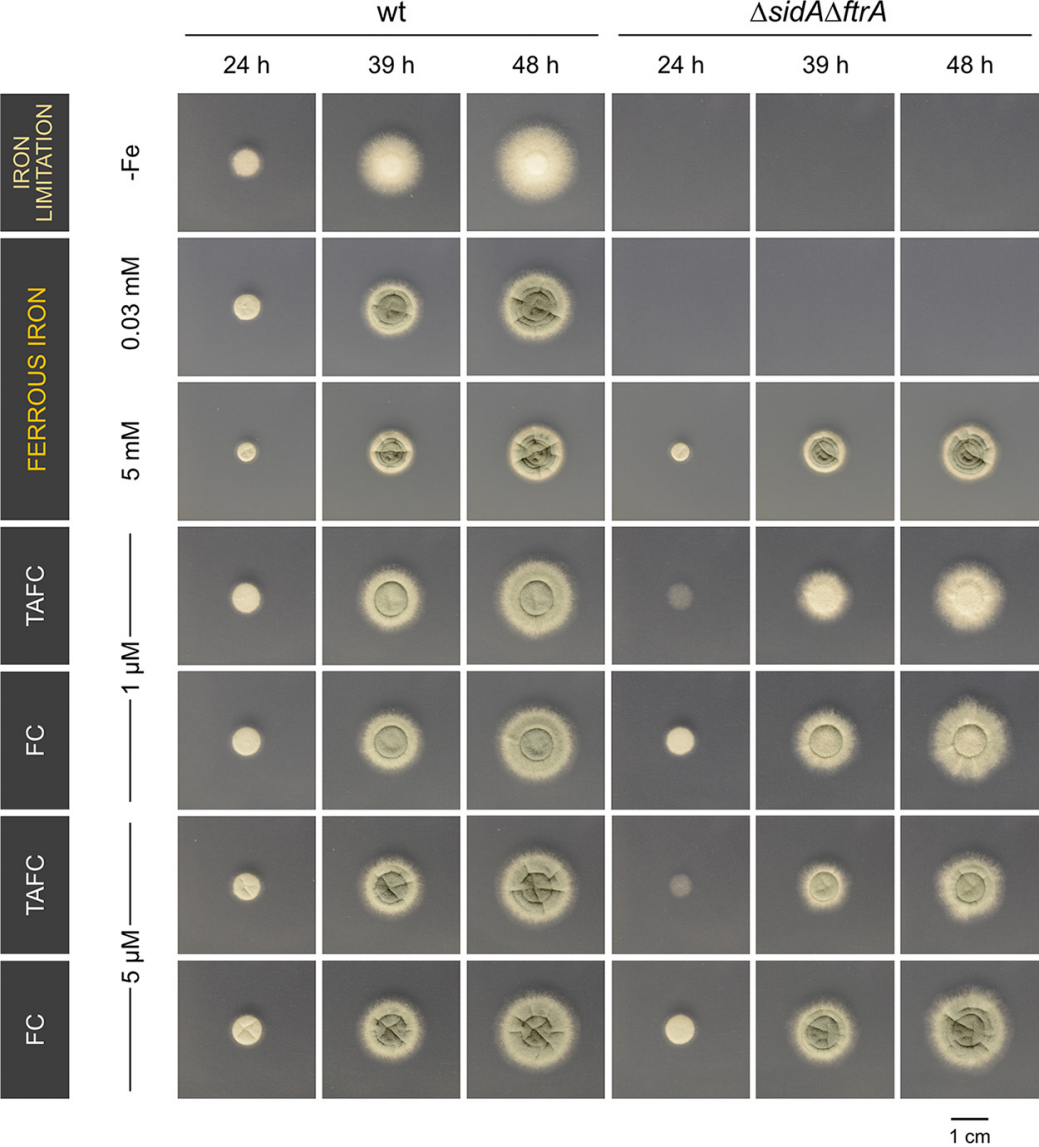
FC promotes colony formation by A. fumigatus Δ*sidAΔftrA* earlier than TAFC. We point inoculated 10^4^ conidia of Aspergillus fumigatus WT and Δ*sidAΔftrA* mutant strains on iron-depleted minimal medium. Plates were incubated at 37°C for 48 h. For supplementation, siderophores were added in the indicated concentration in the ferricrocin form. The green color is indicative of conidiation, as only conidia are greenish pigmented.

In the next step, we aimed to compare growth promotion by TAFC and FC using A. fumigatus WT and the Δ*sidAΔftrA* strain, which is unable to grow without supplementation with >3 mM FeSO_4_ or siderophores utilized by A. fumigatus (10, 35). Radial growth of WT was similar with and without supplementation with iron or the two siderophores, but conidiation was observed only with iron or siderophore supplementation without showing a difference between TAFC and FC (Fig. 10). Sporulation of the Δ*sidAΔftrA* mutant strain was supported significantly better by FC than TAFC, in line with previous studies indicating the importance of FC-mediated iron transport within conidiophores (9, 11). Remarkably, 24 h after inoculation, TAFC promoted only very poor growth compared to FC, even with high TAFC supplementation (5 μM). At later time points, there was still a difference in growth promotion by TAFC and FC, but not as pronounced as at the 24-h time point. The difference between growth promotion by TAFC and FC might reflect the delayed induction of TAFC uptake (MirB) compared to FC uptake (Sit1) during germination (Fig. 2 and 3). Alternatively, chemical differences between TAFC and FC, such as hydrophobicity, might affect the efficiency of growth promotion in the early stage.

## DISCUSSION

So far, FC was shown to play a role in conidial iron storage and intracellular iron transport in A. fumigatus (9, 11). Here, reanalysis of published transcriptome data (30), followed by exemplary confirmation by Northern blotting, revealed that Aspergillus fumigatus exhibits high TLs of genes involved in FC biosynthesis (*sidL*, *sidC*) and FC uptake (*sit1*) in conidia and early germination, which was also confirmed for Sit1 at the protein level by fluorescence monitoring of strains producing Sit1 C-terminally tagged with the yellow fluorescence protein Venus (9, 13, 15, 24). The independence of environmental iron availability of the TL in conidia and during early germination suggests that expression of the genes is subject to developmental, rather than iron regulation in this stage. In contrast, in the late germination phase (early hyphal growth, 6 to 8 h after initiation of germination), the transcript levels of siderophore biosynthetic genes were responsive to the environmental iron availability, suggesting iron regulation. Remarkably, genes involved in TAFC biosynthesis (*sidG*) and uptake (*mirB*) had a very low conidial TLs, and their expression increased only in the late germination phase (9, 20). Taken together, these data indicated a role of FC biosynthesis and uptake in germination, as illustrated in Fig. 1.

In agreement with the extracellular role of FC, FC was identified in the supernatants of liquid-shake culture during germination and hyphal growth by mass spectrometric analyses. Moreover, FC secretion was indicated during growth on solid media using a bioassay based on A. fumigatus mutant strains, the growth of which is (Δ*sidAΔftrA*) or is not (Δ*sidAΔftrAΔsit1AΔsit2*) promoted by FC. In both approaches, FC secretion was independent of the environmental iron availability. Moreover, in the liquid-shake culture, FC secretion started during germination before TAFC secretion, which together might indicate developmental, rather than iron-availability dependent, regulation of FC secretion. At later time points, FC secretion was increased by iron limitation, underlining that, also, this siderophore responds to iron availability during the hyphal phase. Remarkably, TAFC was detected exclusively during iron limitation and 4 h later than FC. These data indicate that TAFC evolved as a purely hyphal siderophore, in contrast to FC, which functions already during germination. At the latest hyphal state analyzed, the 14-h time point, the extracellular TAFC content exceeded that of FC by 400-fold. This difference might explain why extracellular FC has been neglected in previous high-performance liquid chromatography (HPLC)-driven siderophore analyses (9).

Subsequently, growth of a mutant lacking the major fusarinine-type siderophores fusarinine C and TAFC, Δ*sidF* (9), in the presence of BPS, which inhibits siderophore-independent iron acquisition by RIA, or of a mutant lacking both SidF and FtrA, which genetically blocks RIA, under iron limitation strongly indicated that FC mediates high-affinity iron acquisition in A. fumigatus. Moreover, lack of FC uptake by Sit1 was found to decrease the germination rate during iron sufficiency in the absence of RIA, which indicates that germination is fueled by both FC-mediated iron acquisition and RIA. Under iron limitation, both Sit1 and Sit2 were found to be important for germination efficiency, while RIA played a minor role. Noteworthy, simultaneous inactivation of Sit1, Sit2, and even RIA did not affect either radial growth scored after 48 h or biomass formation in liquid media scored after 17 h, indicating that the mutants recover despite the initial germination delay.

Taken together, the data presented here clearly demonstrate FC secretion and its role in iron acquisition. The high conidial TLs in FC-associated genes and FC-mediated iron acquisition during germination reflect the rapid and strong iron demand for cellular proliferation. In other words, FC has a dual role, being secreted for iron acquisition and cellularly accumulated for intracellular iron handling. Such a dual role appears to be not unique, as Ustilago maydis secretes two siderophores, ferrichrome and ferrichrome A, whereby ferrichrome is also found intracellularly (36, 37). Moreover, Schizosaccharomyces pombe produces only a single siderophore, ferrichrome, which is found extra- and intracellularly and has recently been found to be important for spore germination (38, 39).

The presented results are also of translational importance, as Sit1 has recently been shown to mediate uptake of the novel antifungal drug VL-2397, which has a ferrichrome-type structure (24). VL-2397 showed the highest activity during iron limitation, which is most likely caused by the transcriptional repression of Sit1 in the hyphal stage. Nevertheless, VL-2397 showed considerable activity also during iron sufficiency (24), which is most likely mediated by the Sit1 activity during germination shown in this study. Moreover, siderophores showed a promising potential for diagnosis of fungal infections in preclinical studies as well as in clinical samples (40, 41). In an A. fumigatus rat infection model, not only TAFC but also FC was detected in urine and FC also in serum (41). The results of the current study indicate that the detected FC resulted not only from lyses of fungal hyphae but also from active secretion.

In conclusion, this work elucidated the role of FC and its transporters in germination of A. fumigatus. As ferrichrome-type siderophores and Sit1 are highly conserved in mold species (10), the results might be representative of a wide array of species.

## MATERIALS AND METHODS

### Growth conditions.

For spore production, different A. fumigatus strains were cultivated on complete medium (CM) at 37°C, containing 20 g/L glucose (catalog no. HN06.4; Carl Roth GmbH & Co. KG, Karlsruhe, Germany), 2 g/L peptone (catalog no. 8986.1; Carl Roth GmbH & Co. KG), 1 g/L yeast extract (catalog no. MC001; Lab M Limited, Lancashire, UK), 1 g/L Casamino Acids (catalog no. C-0626; Sigma-Aldrich Inc., St. Louis, Missouri, USA), salt solution, and iron-free trace elements (42). For iron-deplete conditions, iron was omitted, and for iron-replete conditions, FeSO_4_ was added as indicated. For Δ*sidA* and Δ*sidAΔftrA* spore production, 1 μM FC was added to CM, and wherever necessary or indicated and for Δ*sidAΔftrAΔsit1Δsit2* strains, 1 μM TAFC was added. All plate growth assays were performed on Aspergillus minimal medium (AMM) containing 1% (w/vol) glucose as carbon source and 20 mM glutamine as nitrogen source (42). For the plate growth assays, 10^4^ spores were point inoculated onto the plates, and for inoculation of liquid media, 10^6^ spores per mL were used; plates and liquid medium were incubated at 37°C. All the Aspergillus fumigatus strains generated and used in this study are listed in Table S2 in the supplemental material.

### Germination assays.

To determine the germination capacity, 10^5^ of the freshly harvested conidia were incubated at 37°C for 8 h in 24-well plates (catalog no. CC7682-7524; CytoOne; Starlab International GmbH, Hamburg, Germany) with 500 μL AMM. For iron-deplete conditions, iron was omitted, and for iron-replete conditions, FeSO_4_ was added to a final concentration of 0.03 mM. The germination of each strain (carried out in triplicates) was scored by light microscopy; 5 random pictures of each well were taken using a reflected-light microscope (Leica DM IL LED; Leica Microsystems GmbH, Wetzlar, Germany) and were counted out. The spores were considered to have germinated when the germ tube was twice as long as the diameter of the spore.

### Detection of Venus-tagged Sit1 during germination.

To determine the level of Venus-tagged Sit1 protein during germination, the previously described strain Sit1^C-Venus^ (Sit1 tagged C-terminally with the yellow fluorescence protein derivative Venus with expression of the tagged *sit1* alleles under the control of the endogenous promoter) (10, 24) was used. The protein level of *sit1^C-Venus^* during germination was measured by inoculating 200 μL of AMM with 5 × 10^6^ conidia and incubating at 37°C for 10 h in a 96-well plate (catalog no. 167008; Nunc MicroWell 96 well; Thermo Fisher Scientific Inc., Waltham, MA, USA). For iron-deplete conditions, iron was omitted, and for iron-replete conditions, FeSO_4_ was added to a final concentration of 0.03 mM. After incubation, fluorescence was measured at 497 ± 15 nm (excitation, 497 ± 15 nm; dichroic mirror, auto, 517.2 nm; emission, 540 ± 20 nm; top excitation and detection) using CLARIOstar Plus microplate reader (BMG Labtech GmbH, Ortenberg, Germany). To visualize the increase of Venus-mediated fluorescence during germination, the unspecific WT background fluorescence of the respective time points, as well as the fluorescence of the 0-h time point, was subtracted from each time point. Three biological triplicates of each reporter strain were analyzed. The mean value and standard deviation were then calculated and displayed.

### Cultivations of the wild-type AfS77 from 0 to 20 h in iron-deficient and iron-sufficient conditions.

To obtain germlings for total RNA extraction and the supernatant of these cultivations, 2.5 × 10^9^ of the freshly harvested conidia was inoculated in 50 mL AMM in 250-mL Erlenmeyer flasks and shaken at 200 rpm at 37°C for 2, 4, 8, 10, 12, and 14 h. For iron-deplete conditions, iron was omitted, and for iron-replete conditions, FeSO_4_ was added to a final concentration of 0.03 mM. After the respective incubation period, the seedlings were harvested, and the supernatant was collected. The germlings were immediately shock-frozen in liquid nitrogen and stored at −80°C, and the supernatant was stored at −40°C.

For dormant conidia (0 h), 1 mL of freshly harvested conidia with a concentration of 3 × 10^9^ was pelleted. The supernatant was discarded, and the pellet was immediately shock-frozen in liquid nitrogen and stored at −80°C until further use.

As a hyphal-stage control, 20-h cultures were grown with 10^6^ conidia/mL in AMM either in iron-depleted or iron-repleted (0.03 mM FeSO_4_) conditions at 37°C and 200 rpm. The mycelium was harvested and lyophilized.

### Northern blot analysis.

The total RNA was isolated either from the cultivated conidia, germlings, and mycelium using TRI reagent (catalog no. T9424; Sigma-Aldrich Inc.) as per the manufacturer’s protocol. A 1.2% agarose gel containing 1.85% (w/vol) formaldehyde was used to separate 10 μg of total RNA. The gel was then blotted onto a Hybond-N+ membrane (Amersham Biosciences, Amersham, UK). For detection by hybridization, digoxigenin-labeled probes amplified by PCR were used. For quantification of Northern blotting data and rRNA, ImageJ/Fiji (v1.53t) (43) was used; transcript levels were normalized to the rRNA of the respective sample (Table S1). The primers used for amplification of the Northern blotting hybridization probes are listed in Table S3.

### Mass spectrometry-based quantification of ferricrocin and TAFC.

The chromatographic purification of the aforementioned supernatants from conidia and germlings, incubated for 2 to 14 h, was carried out according to the method previously described by Oberegger et al. (44) with some modifications listed here. A Pasteur pipette (catalog no. 612-1701; glass Pasteur pipettes; VWR International, Radnor, PA, USA) was filled with Amberlite XAD-16N resin (catalog no. BCCB9752; Sigma-Aldrich Inc.) (swollen in Milli-Q H_2_O) to 3 cm below the rim and rinsed with 5 mL Milli-Q H_2_O. Ten microliters of supernatant from the iron-deplete condition was first saturated with 0.03 mM FeSO_4_ and then applied to the XAD-16N resin column. For iron-replete conditions, 10 mL of the supernatant was directly applied to the XAD-16N resin column. Unspecific bound substances and compounds were removed from the resin by washing it with 10 mL of Milli-Q H_2_O. The bound siderophores were eluted with 2 mL methanol (catalog no. P717.1; Carl Roth GmbH & Co. KG).

The ferricrocin-enriched samples were dried and redissolved in 800 μL of MS-grade MeOH. The liquid chromatography–electrospray ionization–high-resolution mass spectrometry (LC-ESI-HRMS) analysis of the samples was performed on a Vanquish ultra-HPLC (UHPLC) system (Thermo, USA) coupled to an Exploris 120 Orbitrap mass spectrometer (Thermo, USA) using an ESI source. LC parameters were as follows. In the stationary phase, an Accucore C_18_ column, 150 by 2.1 mm and 2.6 μm, was used. The mobile phase consisted of H_2_O (solvent A), and acetonitrile (ACN) (solvent B), with a gradient of 0 min, 4% B; 2 min, 12% B; 15 min, 99% B; and 20 min, stop, and a temperature of 35°C, flow rate of 0.4 mL/min, and injection volume of 1 μL. MS analysis was performed initially on full scan and in positive mode to confirm the molecular weight and also to evaluate the fragmentation pattern of ferricrocin. Mass spectra were acquired between *m/z* 150 and *m/z* 1,500, and full width at half-maximum mass resolution of the Orbitrap mass analyzer was set to 60,000. ESI and global Orbitrap MS parameters were set as follows: spray voltage (positive ion), 3.2 kV; auxiliary gas, 12 U; sheath gas, 45 U; ion transfer tube temperature, 350°C; vaporizer temperature, 400°C; and RF lens, 70%. Quantification of ferricrocin content in the samples was analyzed by means of selected ion monitoring (SIM) experiment. In this regard, full-scan mode analysis of the standard sample revealed two main *m/z*, [M+H]^+^ = 771.24 and [M+Na]^+^ = 793.22. The [M+H]^+^ ion was used for measuring the abundance of its molecular ion peak at *m/z* 771.24. The following parameters were additionally used for SIM analysis: an isolation window (*m/z*) of 1, 1 microscan, and data collected in profile mode. In total, six concentrations (0.00128, 0.0128, 0.128, 1.28, 12.8, and 128 μM) were prepared from the standard stock of ferricrocin (1.28 mM) and analyzed in triplicate (injection volume, 1 μL). The area under the curves obtained were utilized for generating calibration curves. Subsequently, the quantification of ferricrocin in culture supernatants was conducted by analysis of each sample in triplicate (injection volume, 1 μL) and using the previously acquired calibration curves. For a more accurate determination of the concentration of ferricrocin, a standard curve for lower concentrations and one for higher concentrations were generated (Fig. S2). A similar analysis was carried out for quantification of TAFC content in the supernatants. However, only MS1 data were utilized since the corresponding MS peak was well-resolved. To generate calibration curves, ten concentrations (1.11, 2.22, 4.45, 8.90, 17.81, 35.62, 71.25, 142.5, 285, and 570 μg/mL) were prepared in a serial dilution step from a stock of 1.19 mM and injected in triplicate (injection volume, 1 μL). Extracted ion chromatograms (EICs) were utilized for obtaining the area under the curve and subsequently for generation of calibration curves (Fig. S3).

### Generation of A. fumigatus mutant strains.

All mutants generated in this study were performed with A. fumigatus strain AfS77, a derivative of A. fumigatus ATCC 46645 lacking nonhomologous recombination (*akuA::loxP*), except for Δ*sidFΔftrA*, where ATCC 46645 was used as a background strain (45, 46).

For the *sit1* (AFUA_7G06060) deletion in the AfS77 background, genomic DNA from a previously generated Δ*sit1* strain (24) was used to amplify the *sit1* deletion cassette by using oligonucleotides TO102 and TO105. This fragment contains the 5′- and 3′-noncoding region (NCR) of *sit1* and an interjacent *hph* cassette. Following positive confirmation of the *sit1* deletion, the further deletion of *sit2* (AFUA_7G04730) was performed by using plasmid pMA01 (10) containing the Δ*sit2* deletion cassette, which was amplified by using the primers MA01/MA06 (Table S4). Selection for *sit1* deletion transformants was done on AMM containing 0.2 mg/mL hygromycin B (Calbiochem, San Diego, USA). Selection for *sit2* deletion transformants was done on AMM containing 0.1 μg/mL pyrithiamine A (Sigma, Tokyo, Japan). All correct genotypes were confirmed by Southern blotting (Fig. S4).

For generation of plasmid pIH001 containing the *ftrA* (AFUA_5G03800) deletion cassette, four fragments were amplified by PCR, a 1,000-bp-long 5′ NCR of *ftrA* using the oligonucleotides IH009/IH010 for amplification from genomic DNA; a 1,000-bp-long 3′ NCR using IH005/IH006 from genomic DNA; the xylose (*xylP*) promoter-driven expoxidase (*ergA*^P^*^xylP^*) of Penicillium chrysogenum, which imparts resistance to terbinafine (TRB) using IH003/IH004 from plasmid px-*ergA* (47); and pUC19L (Thermo Fisher Scientific Inc.) backbone using IH007/IH008. The generated fragments were then assembled using NEBuilder HiFi DNA assembly reaction (New England Biolabs, Inc. Ipswich, MA, USA). Later, the 5.7-kb-long deletion/knockout cassette was amplified using IH011/IH012, and after purification, the cassette was transformed into Δ*sidF* (A. fumigatus ATCC 46645 background), Δ*sit1*, Δ*sit2*, and Δ*sit1Δsit2* backgrounds. The potential transformants were selected on AMM plates with 0.8 μg/mL TRB (Novartis, Basel, CH) and later confirmed by Southern blotting (Fig. S5).

For generation of the selection marker-free Δ*sidA* A. fumigatus mutant strain in AfS77, *sidA* (AFUA_2G07680) was replaced by a self-excising hygromycin resistance cassette (*hph*) containing the β-rec/six site-specific recombination system under the control of the xylose-inducible promoter (46, 48). Therefore, 1.0 kb of the 5′ and 3′ NCRs of *sidA* were amplified by PCR using the primer pair TO56/TO57 from the previously generated plasmid pΔ*sidA*-rec (10). Selection for *sidA* deletion transformants was done on AMM containing 0.2 mg/mL hygromycin B (Calbiochem), and subsequently, the resistance cassette was excised from Δ*sidA* mutants by cultivation on AMM containing 1% xylose, and correct genotypes were confirmed by Southern blotting (Fig. S6).

For *ftrA* (AFUA_5G03800) deletion in the AfS77 background, genomic DNA from a previously generated Δ*ftrA* ATCC 46645 strain (7) was used to amplify the *ftrA* deletion cassette by using oligonucleotides MA105 and MA106. This fragment contains 5′ and 3′ NCRs of *ftrA* nestling a *hph* cassette. Selection of *ftrA* deletion transformants was done on AMM containing 0.2 mg/mL hygromycin B (Calbiochem), and correct genotypes were confirmed by Southern blotting (Fig. S6).

Transformation of A. fumigatus AfS77 and ATCC 46645 was performed according to Tilburn et al. (49).

### Siderophores.

Ferrioxamine B was purchased from Sigma (Burlington, MA, USA), and ferrioxamine E was purchased from EMC Microcollections GmbH (Tübingen, Germany). The siderophores TAFC and FC used in this study were produced and purified in the laboratory as described by Schrettl et al. and Oberegger et al. (9, 44).

### Transcriptome data set analysis during germination.

The published data set from Baltussen et al. (30) was reanalyzed. The R package DESeq2 was used to obtain the gene count table using A. fumigatus general feature format (GFF) CADRE 30 (50). Normalization of the gene counts (i.e., estimation of size factors and dispersions) was performed with DESeq2 using the default settings. For each time point (0 h, 2 h, 4 h, 6 h, and 8 h) of the two strains used, AfIR964 and AfIR974, the mean of the DESeq2 normalized transcriptome data set was calculated from the two replicates. The figures were visualized using GraphPad Prism version 8.4.3 for Windows (GraphPad Software, San Diego, CA, USA; https://www.graphpad.com/).

### Statistical analysis.

Descriptive statistical analysis was performed with GraphPad Prism version 9.1.0 for Windows (GraphPad Software, San Diego, CA, USA; https://www.graphpad.com/).
