# Supplementary material for: The Siderophore Ferricrocin Mediates Iron Acquisition in Aspergillus fumigatus
Source: Microbiol Spectr. 2023 May 18;11(3):e00496-23. doi: 10.1128/spectrum.00496-23 (PMC10269809; doi:10.1128/spectrum.00496-23)
Supplement: Supplemental file 1 — Supplemental material. Download spectrum.00496-23-s0001.pdf, PDF file, 0.3 MB [file spectrum.00496-23-s0001.pdf]

Table S1 Quantification of Northern blot transcript levels using ImageJ Fiji (v1.53t). The intensity of the transcript levels was normalised to the 28S rRNA. "*" indicates signal saturation.

		+Fe					-Fe				
time [h]	**0**	**2**	**4**	**6**	**8**	**20**	**2**	**4**	**6**	**8**	**20**
sit1	55	70	29	22	21	44	48	24	43	109*	116*
sit2	0	0	0	3	6	76	0	4	9	76	103*
mirB	0	0	0	0	0	0	0	0	0	0	81
sidA	0	0	0	0	0	7	0	0	13	56	56
ftrA	0	0	0	0	4	25	0	0	4	14	63
calA	0	76	122*	39	4	3	80	133*	53	4	75
tubA	0	0	5	48	90	62	0	5	38	87	80
