# Supplementary material for: The Siderophore Ferricrocin Mediates Iron Acquisition in Aspergillus fumigatus
Source: Microbiol Spectr. 2023 May 18;11(3):e00496-23. doi: 10.1128/spectrum.00496-23 (PMC10269809; doi:10.1128/spectrum.00496-23)
Supplement: Supplemental file 2 — Supplemental material. Download spectrum.00496-23-s0002.pdf, PDF file, 0.2 MB [file spectrum.00496-23-s0002.pdf]

Table S2 Strains used in this study.

Strain	Description	Reference
AfS77 (wt)	ATCC4664; *ΔakuA::loxP*	(46)
sit1$^{C\text{-Venus}}$	AfS77; *sit1*::*hph*-p*sit1*-Venus^{C}	(10)
ΔsidF	ATCC46645; *ΔsidF::hph*	(9)
ΔsidAΔftrA	AfS77; *ΔsidA*::*six*, *ΔftrA*::*six*	(10)
ΔsidAΔftrAΔsit1Δsit2	*ΔsidAΔftrA; Δsit1*::*hph, Δsit2*::*ptrA*	(10)
ΔsidFΔftrA	ATCC46645; *ΔsidF::hph; ΔftrA::PxylP:ergA*	This study
ΔsidC	ATCC46645; *ΔsidC::hph*	(9)
ΔsidA	AfS77, *ΔsidA::six*	this study
Δsit1Δsit2	AfS77; *Δsit1::hph, Δsit2::ptrA*	this study
ΔftrA	AfS77; *ΔftrA::hph*	this study
Δsit1Δsit2ΔftrA	*AfS77; Δsit1::hph, Δsit2::ptrA, ΔftrA::PxylP:ergA*	this study
Δsit1ΔftrA	*AfS77; Δsit1::hph, ΔftrA::PxylP:ergA*	this study
Δsit2ΔftrA	*AfS77; Δsit2::ptrA, ΔftrA::PxylP:ergA*	this study
Δsit1	AfS77; *Δsit1::hph*	(24)
Δsit2	AfS77; *Δsit2::ptrA*	(24)
