# Supplementary material for: The Siderophore Ferricrocin Mediates Iron Acquisition in Aspergillus fumigatus
Source: Microbiol Spectr. 2023 May 18;11(3):e00496-23. doi: 10.1128/spectrum.00496-23 (PMC10269809; doi:10.1128/spectrum.00496-23)
Supplement: Supplemental file 3 — Supplemental material. Download spectrum.00496-23-s0003.pdf, PDF file, 0.2 MB [file spectrum.00496-23-s0003.pdf]

Table S3 Primer used for the amplification of the digoxigenin-labelled probes for Northern- and Southern blot analysis.

Probe	Gene	5' - 3' Sequence
sit1-CDS	AFUA_7G06060	AGAACCAACCATGAACATGGCGATGCAC<br>TTACGGATGATTTATCATCAATCTCCTCCG
sit2-CDS	AFUA_7G04730	GGGTTCTGCTTGTTCTTTGC<br>CCGCAATGGCAGGGATTCCC
mirB-CDS	AFUA_3G03640	AAGCCGAGAAAAAGGGGG<br>AACCCAGATGAAGCCCAG
mirC-CDS	AFUA_2G05730	TTGGTTGTCCAGAATGTGGTTAACG<br>CACGAGGCGTGCTTCAGC
sidA-CDS	AFUA_2G07680	AACTACCTCCACCAGAAG<br>GAACGGCAATGTTGTAAG
ftrA-CDS	AFUA_5G03800	ATGGCAAAAGACGTATTTGC<br>TCAGACAAGGGATGCTC
calA-CDS	AFUA_3G09690	CAAACAACACTCTCTTGAC<br>CCAGGAGTGAGAGAATG
tubA-CDS	AFUA_1G10910	ATATGTTCCTCGTGCCGTTC<br>CCTCAGTGAACTCCATCTC
3' NCR *sit1*	AFUA_7G06060	TTTGATAATAGGCAAACGAC<br>GCTCGGTCAGAAAGTCG
5' NCR *sit2*	AFUA_7G04730	AGCTCTTCATTGTCGCAAG<br>CATGCTCGAGAAACCAATG
3' NCR *ftrA*	AFUA_5G03800	AAACTCTTCACCCAGCCAGC<br>AGCGTCATTTCTGCGCCA
3' NCR *sidA*	AFUA_2G07680	CACTGCTTCTGACTATC<br>TTCCAGGTGGAAGCAAG
