# Supplementary material for: The Siderophore Ferricrocin Mediates Iron Acquisition in Aspergillus fumigatus
Source: Microbiol Spectr. 2023 May 18;11(3):e00496-23. doi: 10.1128/spectrum.00496-23 (PMC10269809; doi:10.1128/spectrum.00496-23)
Supplement: Supplemental file 4 — Supplemental material. Download spectrum.00496-23-s0004.pdf, PDF file, 0.2 MB [file spectrum.00496-23-s0004.pdf]

Table S4 Primers used in this study.

Primer	5’ - 3’ Sequence
IH003	gactcacaagCGACGGAAGCGCGCAGTC
IH004	ggtcactcgtGTAGACTTTCCTCAGAGCCTTTTTGTTGTC
IH005	gaaagtctacACGAGTGACCCCCAAAGAG
IH006	cttgcatgccGTTGATTGCAGAAGTCTTATTGTCG
IH007	tgcaatcaacGGCATGCAAGCTTGGCGT
IH008	tgcctgagacGTACCGAGCTCGAATTCACTG
IH009	agctcggtacGTCTCAGGCAATTTCAACCC
IH010	gcttccgtcgCTTGTGAGTCGCGAGGGAG
IH011	GTCTCAGGCAATTTCAACCCG
IH012	GAAGTCTTATTGTCGGGTGAGA
TO102	AAGCTCGTCCCCTCCAG
TO105	GCTCGGTCAGAAAGTCG
MA01	AATTCGAGCTCGGTACCTCCGTTGTCCAGGGTCAGTACAG
MA06	GCCAAGCTTGCATGCCGGTTGGCCTGCAACGAGGCTTGTC
TO56	TGCGCACAAAAGAGGACG
TO57	CACACTGCTTCTGACTATC
MA105	TTCTGCGTGTCGTTCATCAGA
MA106	AAGTTGAGACTTTGGCCGTA
