# Supplementary material for: The Siderophore Ferricrocin Mediates Iron Acquisition in Aspergillus fumigatus
Source: Microbiol Spectr. 2023 May 18;11(3):e00496-23. doi: 10.1128/spectrum.00496-23 (PMC10269809; doi:10.1128/spectrum.00496-23)
Supplement: Supplemental file 5 — Supplemental material. Download spectrum.00496-23-s0005.pdf, PDF file, 0.4 MB [file spectrum.00496-23-s0005.pdf]

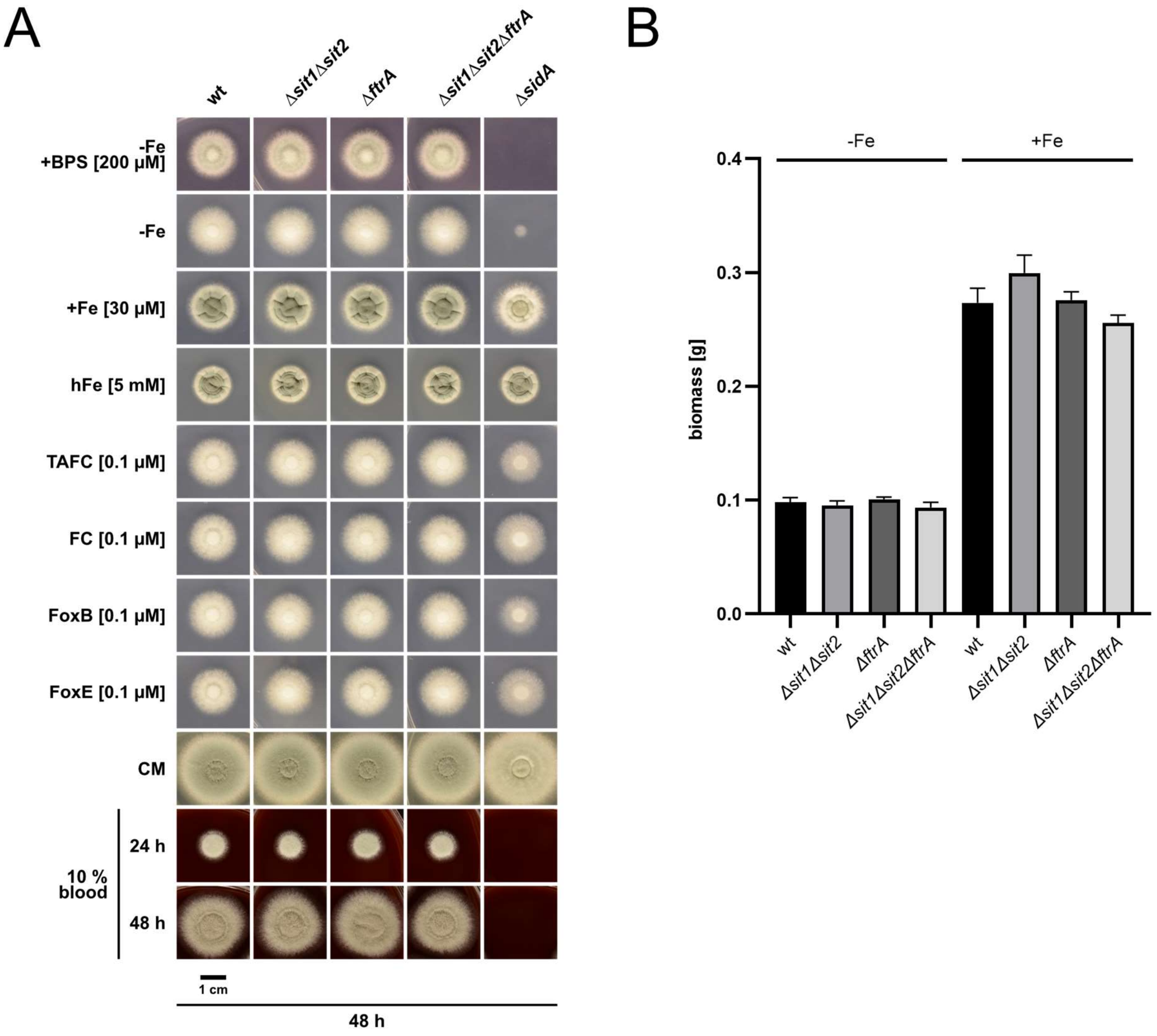

FIG S1 The mutants *Δsit1Δsit2*, *ΔftrA,* and *Δsit1Δsit2ΔftrA* display wt-like radial growth on solid media (A) and biomass formation in liquid media (B). (A) 10^4 conidia of *Aspergillus fumigatus* wt and mutant strains were point-inoculated on media described in legend of Fig. 7 as well as on AMM supplemented with 5mM $FeSO_4$ (hFe) or 0.1 µM ferric siderophores (TAFC; FC; FoxB, ferrioxamine B; FoxE, ferrioxamine E) as well as on complete medium (CM). Plates were incubated at 37 °C for 48 h. (B) 10^8 conidia of *Aspergillus fumigatus* wt and mutant strains were cultivated in 100 mL AMM without (-Fe) or with (+Fe) addition of $FeSO_4$ to a final concentration of 0.03 mM, and shaken with 200 rpm at 37 °C for 17 h. Shown are the mean values ± SD of biological triplicates.
