# Supplementary material for: The Siderophore Ferricrocin Mediates Iron Acquisition in Aspergillus fumigatus
Source: Microbiol Spectr. 2023 May 18;11(3):e00496-23. doi: 10.1128/spectrum.00496-23 (PMC10269809; doi:10.1128/spectrum.00496-23)
Supplement: Supplemental file 6 — Supplemental material. Download spectrum.00496-23-s0006.pdf, PDF file, 0.2 MB [file spectrum.00496-23-s0006.pdf]

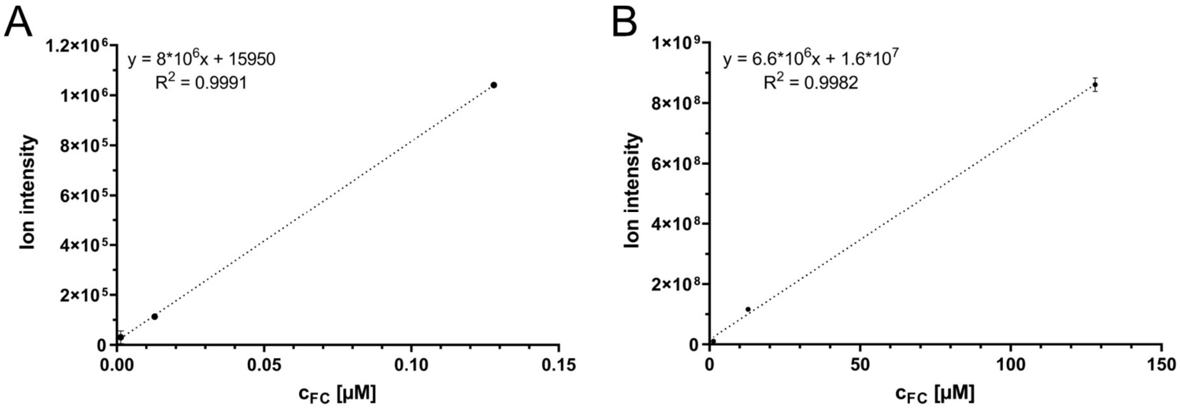

FIG S2 The standard lines for calculating the concentration of FC during germination. (A) Is the standard line for determining the concentration in the low concentration range. $y = 8*10^6x + 15950$; $R^2 = 0.9991$. (B) Is the standard line for determining the concentration in the higher concentration range. $y = 6.6*10^6x + 1.6*10^7$; $R^2 = 0.9982$.
