# Supplementary material for: The Siderophore Ferricrocin Mediates Iron Acquisition in Aspergillus fumigatus
Source: Microbiol Spectr. 2023 May 18;11(3):e00496-23. doi: 10.1128/spectrum.00496-23 (PMC10269809; doi:10.1128/spectrum.00496-23)
Supplement: Supplemental file 7 — Supplemental material. Download spectrum.00496-23-s0007.pdf, PDF file, 0.2 MB [file spectrum.00496-23-s0007.pdf]

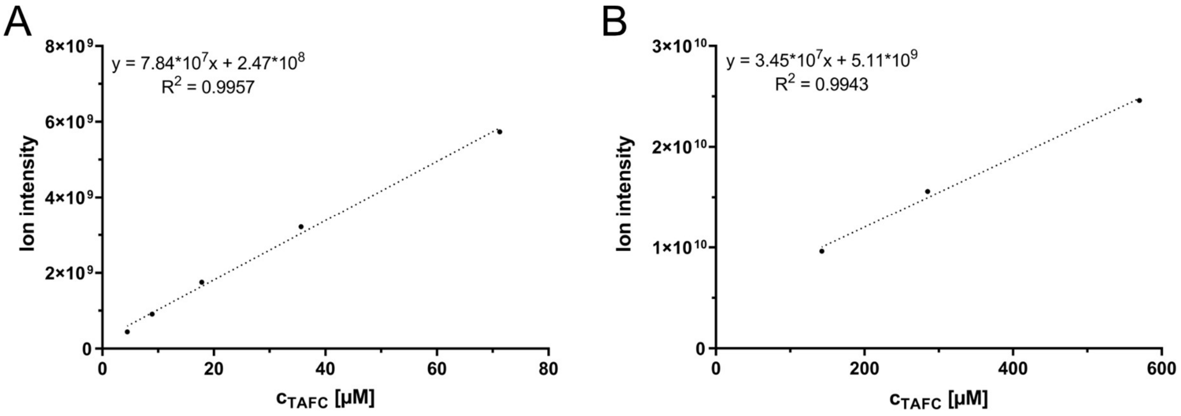

FIG S3 The standard lines for calculating the concentration of TAFC during germination. (A) Is the standard line for determining the concentration in the low concentration range. $y = 7.84*10^7x + 2.47*10^8$; $R^2 = 0.9957$. (B) Is the standard line for determining the concentration in the higher concentration range. $y = 3.45*10^7x + 2.47*10^8$; $R^2 = 0.9943$.
