# Supplementary material for: The Siderophore Ferricrocin Mediates Iron Acquisition in Aspergillus fumigatus
Source: Microbiol Spectr. 2023 May 18;11(3):e00496-23. doi: 10.1128/spectrum.00496-23 (PMC10269809; doi:10.1128/spectrum.00496-23)
Supplement: Supplemental file 8 — Supplemental material. Download spectrum.00496-23-s0008.pdf, PDF file, 0.3 MB [file spectrum.00496-23-s0008.pdf]

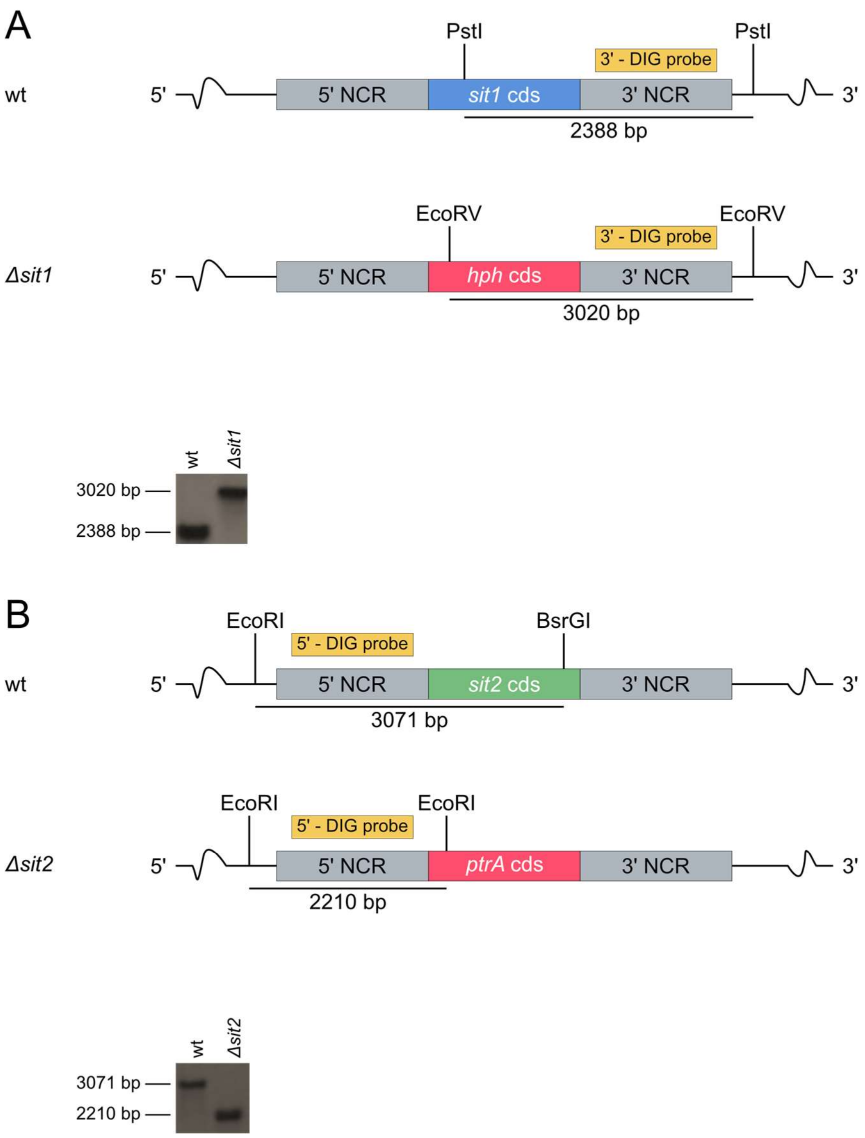

FIG S4 Deletion scheme of *sit1 and sit2* gene in the *A. fumigatus* wild-type AfS77. (A) Genomic schematic map of the *sit1* locus in wt and *Δsit1*. Digestion with the restriction enzyme PstI resulted in a fragment of 2388 bp for wt and digestion with EcoRV resulted in a fragment of 3020 bp for *Δsit1*. (B) Genomic schematic map of the *sit2* locus in wt and *Δsit2*. Joint digestion with the restriction enzymes BsrGI and EcoRI resulted in a fragment of 3071 bp in wt and a fragment length of 2210 bp when *sit2* has been deleted.
