# Supplementary material for: The Siderophore Ferricrocin Mediates Iron Acquisition in Aspergillus fumigatus
Source: Microbiol Spectr. 2023 May 18;11(3):e00496-23. doi: 10.1128/spectrum.00496-23 (PMC10269809; doi:10.1128/spectrum.00496-23)
Supplement: Supplemental file 9 — Supplemental material. Download spectrum.00496-23-s0009.pdf, PDF file, 0.4 MB [file spectrum.00496-23-s0009.pdf]

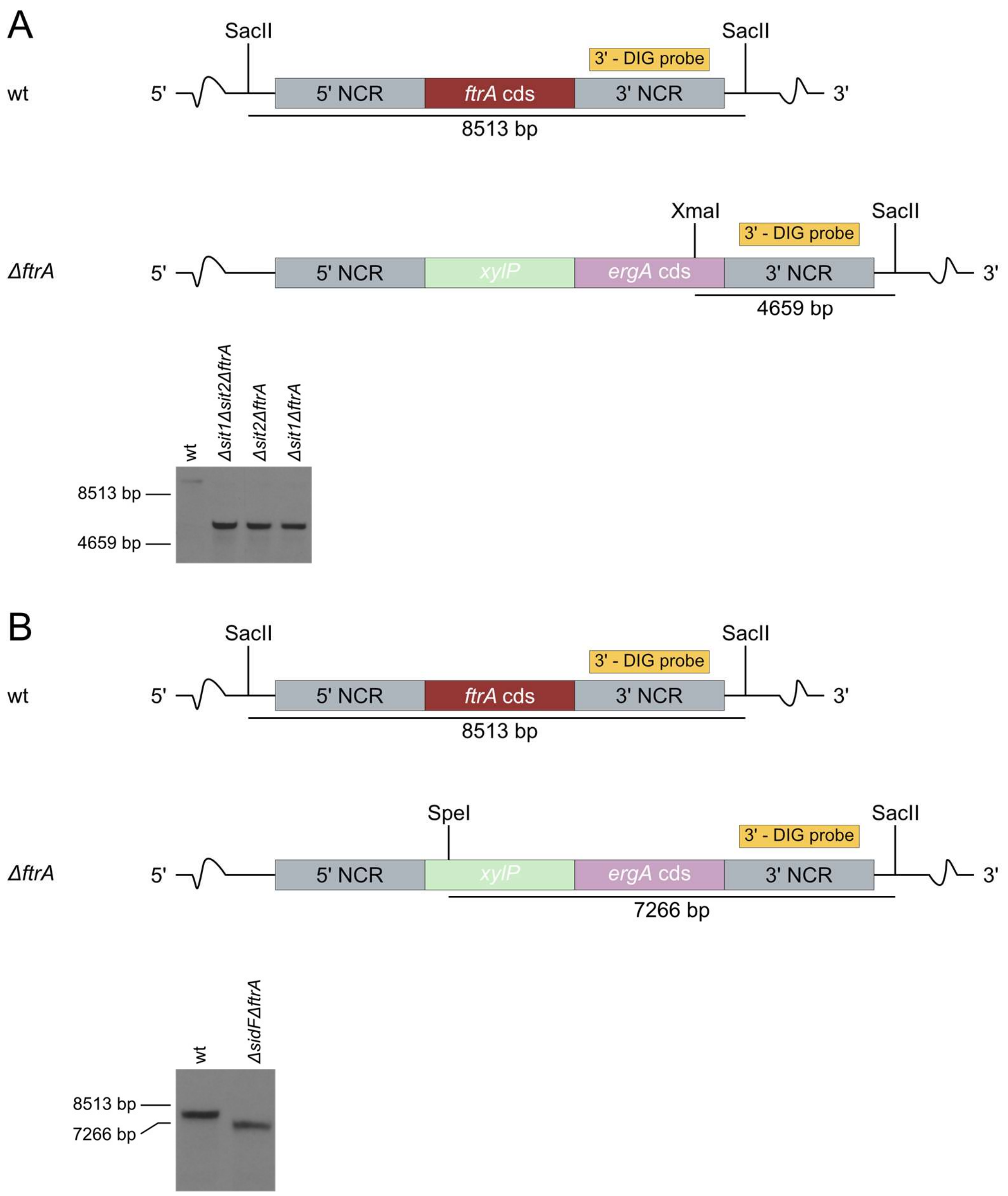

FIG S5 Deletion scheme of *ftrA* gene in the *Δsit1*, *Δsit2, Δsit1Δsit2* and *ΔsidF* mutation strains of *A. fumigatus*. (A) Genomic schematic map of the *ftrA* locus in wt and *ΔftrA*. Joint digestion with the restriction enzymes SacII and XmaI resulted in a fragment of 8513 bp in wt and a fragment length of 4659 bp when *ftrA* has been deleted. (B) Genomic schematic map of the *ftrA* locus in wild-type AfS77 and *ΔftrA*. Joint digestion with the restriction enzymes SacII and SpeI resulted in a fragment of 8513 bp in wt and a fragment length of 7266 bp when *ftrA* has been deleted.
