# Supplementary material for: The Siderophore Ferricrocin Mediates Iron Acquisition in Aspergillus fumigatus
Source: Microbiol Spectr. 2023 May 18;11(3):e00496-23. doi: 10.1128/spectrum.00496-23 (PMC10269809; doi:10.1128/spectrum.00496-23)
Supplement: Supplemental file 10 — Supplemental material. Download spectrum.00496-23-s0010.pdf, PDF file, 0.4 MB [file spectrum.00496-23-s0010.pdf]

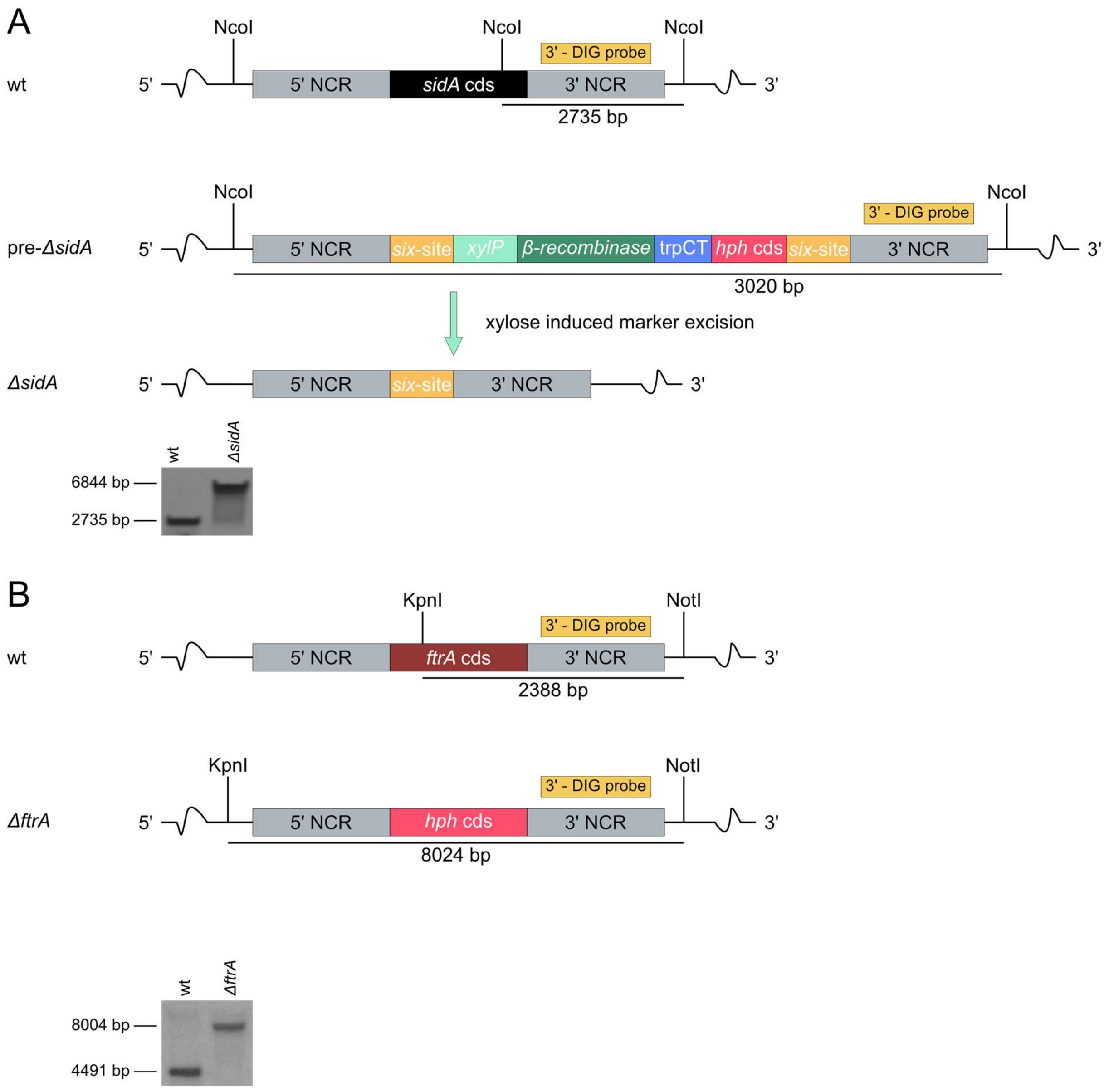

FIG S6 Deletion scheme of *sidA* and *ftrA* gene in *A. fumigatus*. (A) Genomic schematic map of the *sidA* locus in wt and *ΔsidA*. Digestion with the restriction enzyme NcoI resulted in a fragment of 6844 bp in wt and a fragment length of 2735 bp when *sidA* has been deleted. (B**)** Genomic schematic map of the *ftrA* locus in wt and *ΔftrA*. Joint digestion with the restriction enzymes KpnI and NotI resulted in a fragment of 8004 bp in wt and a fragment length of 4491 bp when *ftrA* has been deleted.
